# CD36^+^ cancer-associated fibroblasts provide immunosuppressive microenvironment for hepatocellular carcinoma via secretion of macrophage migration inhibitory factor

**DOI:** 10.1038/s41421-023-00529-z

**Published:** 2023-03-06

**Authors:** Gui-Qi Zhu, Zheng Tang, Run Huang, Wei-Feng Qu, Yuan Fang, Rui Yang, Chen-Yang Tao, Jun Gao, Xiao-Ling Wu, Hai-Xiang Sun, Yu-Fu Zhou, Shu-Shu Song, Zhen-Bin Ding, Zhi Dai, Jian Zhou, Dan Ye, Duo-Jiao Wu, Wei-Ren Liu, Jia Fan, Ying-Hong Shi

**Affiliations:** 1 Department of Liver Surgery and Transplantation, Liver Cancer Institute, Zhongshan Hospital, Fudan University; Key Laboratory of Carcinogenesis and Cancer Invasion of Ministry of Education, Shanghai, China; 2 Research Unit of Liver cancer Recurrence and Metastasis, Chinese Academy of Medical Sciences, Beijing, China; 3 Department of Immunology and Pathogenic Biology, School of Basic Medical Sciences, Shanghai University of Traditional Chinese Medicine, Shanghai, China; 4 Department of Biochemistry and Molecular, School of Basic Medical Sciences, Fudan University, Shanghai, China; 5 Shanghai Key Laboratory of Medical Epigenetics, International Co-laboratory of Medical Epigenetics and Metabolism (Ministry of Science and Technology), and Key Laboratory of Metabolism and Molecular Medicine (Ministry of Education), and Molecular and Cell Biology Lab, Institutes of Biomedical Sciences, Shanghai Medical College of Fudan University, Shanghai, China; 6 Institute of Clinical Science, Zhongshan Hospital, Fudan University, Shanghai, China

**Keywords:** Cancer microenvironment, Tumour heterogeneity, Cancer immunotherapy

## Abstract

Hepatocellular carcinoma (HCC) is an immunotherapy-resistant malignancy characterized by high cellular heterogeneity. The diversity of cell types and the interplay between tumor and non-tumor cells remain to be clarified. Single cell RNA sequencing of human and mouse HCC tumors revealed heterogeneity of cancer-associated fibroblast (CAF). Cross-species analysis determined the prominent CD36^+^ CAFs exhibited high-level lipid metabolism and expression of macrophage migration inhibitory factor (MIF). Lineage-tracing assays showed CD36^+^CAFs were derived from hepatic stellate cells. Furthermore, CD36 mediated oxidized LDL uptake-dependent MIF expression via lipid peroxidation/p38/CEBPs axis in CD36^+^ CAFs, which recruited CD33^+^myeloid-derived suppressor cells (MDSCs) in MIF- and CD74-dependent manner. Co-implantation of CD36^+^ CAFs with HCC cells promotes HCC progression in vivo. Finally, CD36 inhibitor synergizes with anti-PD-1 immunotherapy by restoring antitumor T-cell responses in HCC. Our work underscores the importance of elucidating the function of specific CAF subset in understanding the interplay between the tumor microenvironment and immune system.

## Introduction

Hepatocellular carcinoma (HCC) is the fourth leading cause of cancer-related death worldwide, and chronic hepatitis B (HBV) virus infection is the leading risk factor^1^. The recurrence rate after surgical resection remains high, and the prognosis is still poor for patients with unresectable HCC and treatment options are limited^1^. More than 80% of HCC cases are characterized by extensive liver fibrosis caused by the activation, proliferation, and accumulation of fibroblasts^2^. A hallmark feature of the tumor microenvironment (TME) of HCC is the mass of cancer-associated fibroblasts (CAFs), which can secrete multiple cytokines, chemokines, and growth factors, supporting cancer cells both directly and indirectly^2,3^. Although these CAF-derived factors serve as direct survival signals to cancer cells, they also alter the immune cell milieu by inhibiting the activity of immune effector cells and recruiting immune suppressor cells, allowing cancer cells to evade immune surveillance^4^.

In previous studies, different CAF subtypes with various functions have been extensively revealed in human pancreatic ductal adenocarcinoma (PDAC)^5^, head and neck squamous cell carcinomas^6^, breast cancer^7^ and lung tumors^8^. Hence, the multifaceted nature of CAFs suggests that they include diverse subpopulations, and an improved understanding of stromal heterogeneity may explain how CAFs contribute to the dynamic complexity and functional malleability of the tumor ecosystem^3^. Recent advances in single-cell RNA sequencing (scRNA-seq) have overcome some of the technical hurdles in the investigation of cellular heterogeneity among complex tissues such as carcinomas^9,10^. Specifically, scRNA-seq has been applied to segregate CAFs into inflammatory (iCAF) and myofibroblastic (myCAF) subpopulations in intrahepatic cholangiocarcinoma (ICC) that display distinct ligand-receptor interactions and promote ICC via different CAF-cancer cell interactions^11,12^. However, stromal heterogeneity, especially CAFs, and the interplay between malignant cells and stromal cells at single-cell resolution in HCC remain poorly understood. Although previous studies have reported that CAFs modulate HCC progression through various mechanisms regardless of the CAF subtypes within tumors^4^, it is unclear whether different protumoral mechanisms underlying CAF subpopulations exist in HCC, and the intricate crosstalk between CAFs and other components in the TME remains unknown, particularly for different CAF subtypes.

Here, we performed scRNA-seq of human or murine HCC tumors and identified 5 common CAF subtypes in HCC tumors, namely, vCAFs, mCAFs, lpmCAFs (CD36^+^ CAFs), lpCAFs and apCAFs, among which the newly identified CD36^+^ CAF subset was highlighted. CD36 mediated oxidized LDL uptake-dependent migration inhibitory factor (MIF) expression via the lipid peroxidation/p38/CEBPs axis in CD36^+^ CAFs, which recruited CD33^+^ MDSCs in a macrophage MIF- and CD74-dependent manner. Moreover, CD36^+^ CAFs potentiated the capacity of MDSCs to enhance the immunosuppressive TME and cancer stemness. The number of CD36^+^ CAFs might predict the HCC immunotherapy response, and targeting CD36 with SSO was used to synergistically enhance the efficacy of immunotherapy in different murine HCC models. Together, our results provide a comprehensive transcriptomic overview and reveal novel intercellular crosstalks between CD36^+^ CAFs, CD33^+^ MDSCs and HCC cells, suggesting a potential microenvironment-targeting combination therapy for HCC.

## Results

### Single-cell analysis uncovers the complexity of human HCC tumors

**Fig. 1:**
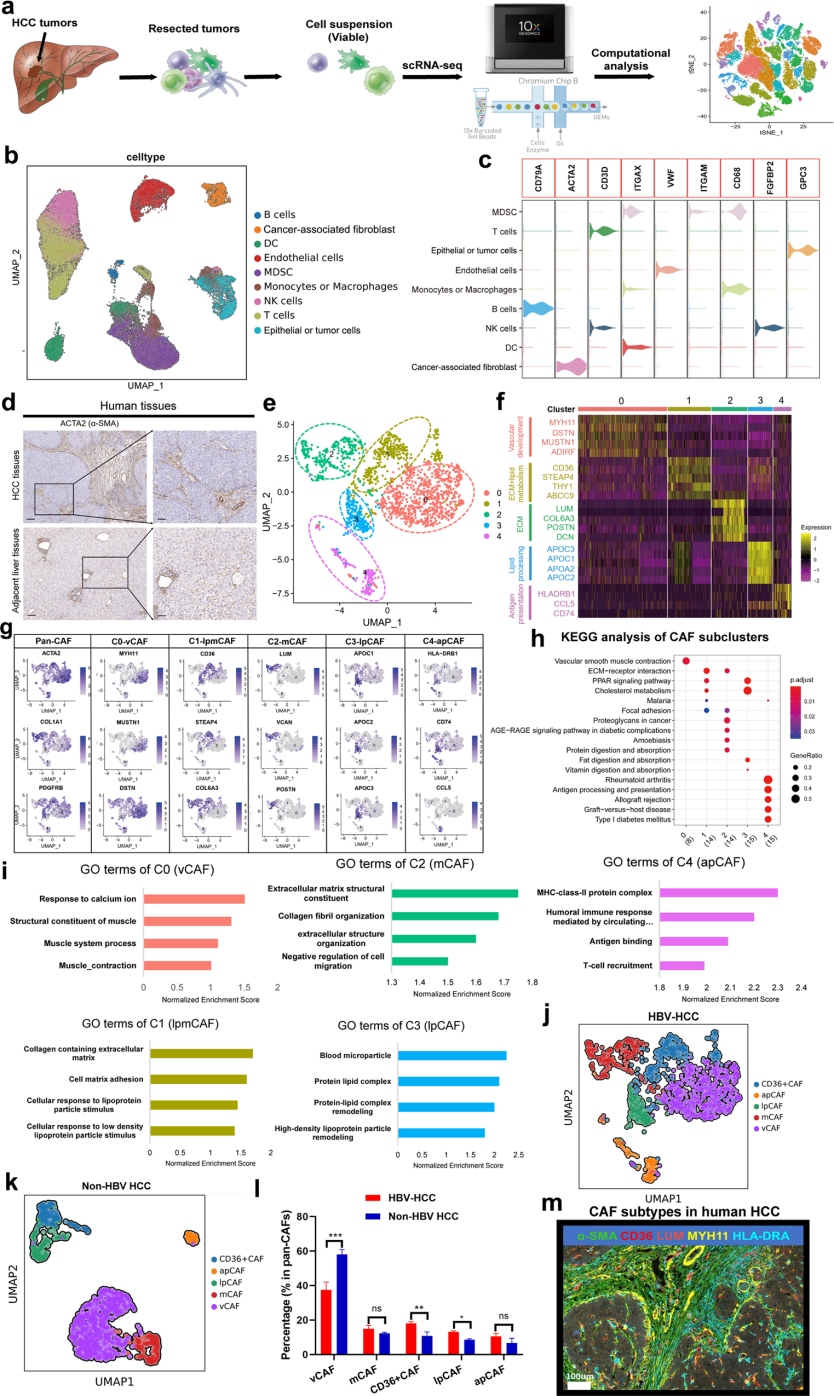
Distinct fibroblast subpopulations in human HBV-related HCC. **a** Schematic diagram of scRNA-seq analysis workflow. HCC and adjacent tissues were dissociated into single cells, and sequenced using 10× Genomics platform. **b** UMAP plots for the different clusters’ identification of single cells in human HCC tumor. **c** Violin plots showing the specific cell marker for different cell types in human HCC tissues. **d** α-SMA expression in human HCC and normal tissues by IHC experiments. **e** UMAP plots show 5 different CAF clusters in human HCC tumors. **f** Heatmap showing the top DEGs (Wilcoxon test) in each CAF subtypes. **g** UMAP plots show marker genes in different CAF clusters. **h** KEGG analysis in different CAF subtypes. **i** GO analysis in different CAF subtypes. **j** UMAP plots for CAF subtypes in HBV related HCC tumors. **k** UMAP plots for CAF subtypes in non-HBV related HCC tumors. **l** The comparison of CAF subtypes between HBV-HCC and non-HBV HCC tumors. **m** Multiplex immunofluorescence staining showed major CAF clusters existed in human HCC tissues.

To comprehensively characterize the populations of cells that are present in human HCC, we undertook an scRNA-seq approach to transcriptionally characterize a large number of cells in primary tumors. Seven tumors from patients with untreated HBV-related HCC and adjacent liver tissues from four of these patients (Supplementary Table S1) were enzymatically digested to generate single-cell suspensions (Fig. 1a; 110658 cells). After stringent filtering, 90572 cells were retained for further analysis. Following gene expression normalization, we conducted dimensionality reduction and clustering using principal component analysis and a uniform manifold approximation and projection (UMAP), respectively (Fig. 1b and Supplementary Fig. S1a). Thereafter, copy number variation (CNV) analysis was employed to distinguish malignant and nonmalignant cells (Supplementary Fig. S1b, c). The cells could be assigned to 9 distinct cell types (Fig. 1b) using known marker genes: epithelial and tumor cells (5059 cells, 5.6%, marked with *EPCAM*, *ALDH1A1* and *ALB*); B cells (1332 cells, 1.5%, marked with *MS4A1* and *CD79A*); T cells (24895 cells, 27.5%, marked with *CD3D* and *CD3E*); natural killer (NK) cells (13868 cells, 15.3%, marked with *FGFBP2* and *FCG3RA*); myeloid-derived suppressor cells (MDSCs) (11087 cells, 12.2%, marked with *ITGAM* and *CD33*); monocytes or macrophages (9978 cells, 11.0%, marked with *CD68*, *CD163*, and *CD14*); dendritic cells (7784 cells, 8.6%, marked with *ITGAX*); fibroblasts (2495 cells, 2.8%, marked with *ACTA2* and *COL1A2*); and endothelial cells (14074 cells, 15.5%, marked with *PECAM1* and *vWF*; Fig. 1c and Supplementary Fig. S1d, e, Table S2). Remarkably, epithelial and tumor cells and other cells, including MDSCs, T cells, DCs and CAFs were highly patient specific, suggesting prominent molecular intertumoral heterogeneity among HCC samples (Supplementary Fig. S1f). The differentially expressed genes (DEGs) and marker genes, as shown in the dot plots, confirmed the accuracy of cell identity (Fig. 1c and Supplementary Fig. S1d, e).

### Identification and molecular characterization of five CAF subtypes in human HCC

More than 80% of HCC cases are characterized by extensive liver fibrosis caused by the activation and accumulation of fibroblasts^2^, as demonstrated by α-SMA immunohistochemistry (IHC) staining (Fig. 1d). We then sorted 1835 CAFs in our scRNA-seq analyses derived from 7 HBV-related HCC tissues, and the cells were clustered into 5 subpopulations (Fig. 1e). All 5 subclusters expressed high levels of canonical fibroblast markers, such as *ACTA2* (α-SMA), *COL1A2*, and *COL1A1*, confirming their identity as fibroblasts (Supplementary Fig. S1g); however, each subcluster displayed distinct transcriptomic signatures (Fig. 1f, g and Supplementary Fig. S1g, h).

Subcluster 0 CAFs accounted for the majority of the CAF populations (40%) and were characterized by signature microvasculature genes, such as *MYH11*, *MUSTN1*, and *MCAM* (Fig. 1f, g and Supplementary Fig. S1g). Thus, we designated them vascular CAFs (vCAFs, vCAFs-c0-MYH11). Gene ontology (GO) and KEGG analyses of vCAFs indicated significant enrichment in vascular smooth muscle contraction and response to calcium ions, consistent with their microvascular signatures (Fig. 1h, i). Subcluster 2 CAFs expressed low levels of α-SMA and high levels of extracellular matrix (ECM) signatures, including collagen molecules (*COL5A1* and *COL6A3*), periostin (*POSTN*), LUM, DCN and FAP (Fig. 1f, g and Supplementary Fig. S1h). Interestingly, the GO terms enriched in this subtype were associated with ECM and collagen fibril organization. Hence, we accordingly designated them as matrix CAFs (mCAFs, mCAFs-c2-LUM, Fig. 1h, i). KEGG analysis showed that the epithelial to mesenchymal transition (EMT) pathway was highlighted in mCAFs (Supplementary Fig. S1i). Similar to mCAFs-c2-LUM, subcluster 1 CAFs also expressed high levels of *COL6A3* and *COL1A1* as well as high levels of lipid metabolism-related genes (*CD36* and *STEAP4*, Fig. 1f, g and Supplementary Fig. S1h). Additionally, the GO and KEGG terms enriched for this subcluster were related to ECM, cholesterol metabolism, hallmarks of fatty acid metabolism and reactive oxygen species (ROS) pathways (Fig. 1h, i and Supplementary Fig. S1j, k), indicating that this subcluster may engage in both ECM and cholesterol metabolism. Accordingly, CAFs in this subcluster were named lipid processing (lp)-mCAFs (lpmCAFs, lpmCAFs-c1-CD36; Fig. 1f, g). Subcluster 3 CAFs expressed high levels of lipid processing markers, including APOA1 and APOC1 (Fig. 1g and Supplementary Fig. S1g). Interestingly, GO and KEGG analyses of these CAFs indicated significant enrichment in protein-lipid complex remodeling and hallmark of fatty acid metabolism; therefore, we termed them lipid-processing CAFs (lpCAFs, lpCAFs-c3-APOA1; Fig. 1h, i). Consistent with a previous report concerning human PDAC^5^ and ICC tumors^12^, we found that subcluster 4 CAFs expressed major histocompatibility complex II (MHCII) genes, such as *CD74* and *HLA-DRA*, as well as chemokine-related genes, such as *CCL5* (Fig. 1f, g and Supplementary Fig. S1h). Moreover, the GO terms and KEGG pathways enriched in this subcluster were related to the MHC-class-II protein complex and antigen processing and presentation (Fig. 1h, i and Supplementary Fig. S1l); therefore, we termed them antigen-presenting CAFs (apCAFs, apCAFs-c4-HLA-DRA). Finally, to explore the heterogeneity of CAF subtypes caused by different etiologies, we performed scRNA-seq of 7 non-HBV-related HCC tumors and found that the proportions of CD36^+^ CAFs and lpCAFs were significantly decreased in non-HBV HCC (including alcohol-, fatty liver- or drug-induced HCC) compared with HBV-HCC (Fig. 1j–l and Supplementary Fig. S1m, n), which may be caused by HBV protein-induced lipid metabolism and oxidative stress in HBV-related HCC^13^. Finally, we confirmed the presence of the main CAF subsets in human HCC samples using multiplex immunofluorescence (mIF) staining (Fig. 1m).

### Single-cell analysis of CAF subtypes in mouse HCC tumors recapitulates the subtypes found in human HCC

**Fig. 2:**
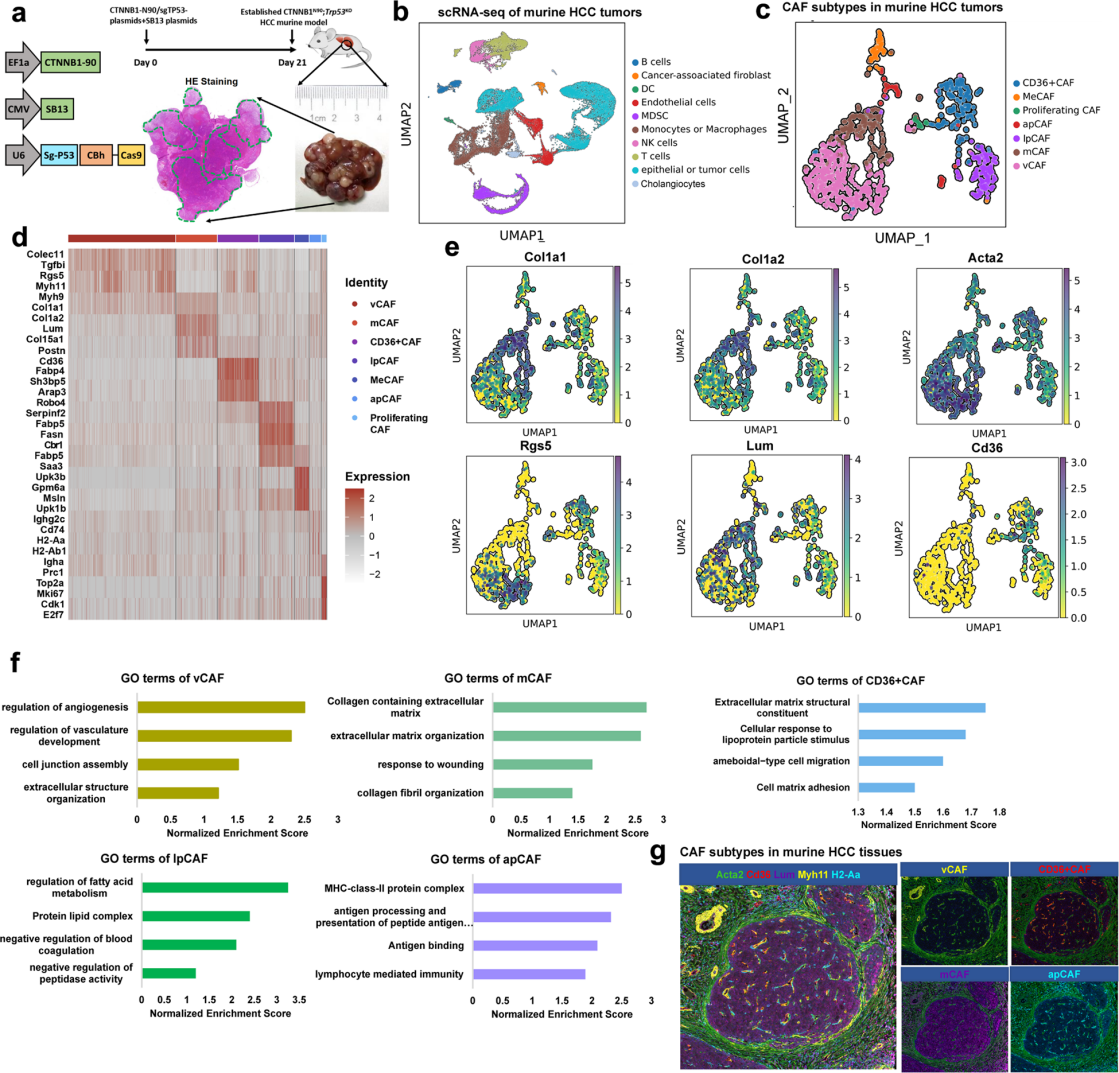
Distinct fibroblast subpopulations in murine HCC tissues. **a** Schematic diagram of established HCC murine model by HDTVi. **b** UMAP plots showing different cell types from scRNA-seq data in murine HCC tissues. **c** UMAP plots showing CAF subtypes in murine HCC tissues. **d** Heatmap showing the top DEGs (Wilcoxon test) in each cell types in murine HCC tumors. **e** UMAP plots for each marker genes in murine CAF subtypes. **f** GO analysis in different murine CAF subtypes. **g** Multiplex immunofluorescence staining showed major CAF clusters existed in murine HCC tissues.

Our results in human HCC specimens confirmed the presence of five CAF subclusters. To allow deeper CAF characterization, we extended our investigation to a murine HCC model. Our previous study showed that *CTNNB1* (58%) and *TP53* (19%) were ranked as the two most mutated genes in our cohort of HBV-related HCC patients^14^, which was further validated in TCGA HCC database (Supplementary Fig. S2a). Therefore, we performed hydrodynamic tail-vein injection (HDTVi) of px330-sg-p53 and CMV-SB13 combined with CTNNB1-N90 into 6-week-old C57BL/6J mice to establish *CTNNB1*^*N90*^;*Trp53*^*KO*^ HCC murine models to mimic the HCC genetic background (Fig. 2a)^15,16^. Similar to human HCC, our Acta2-IHC experiment showed a large accumulation of fibroblasts in murine HCC tumors (Supplementary Fig. S2b). Based on this model, seven tumors from HCC mice were dissociated into single cells, for a total of 63977 viable cells after stringent filtering (Fig. 2b). Clustering analysis of this dataset resulted in 10 clusters, with a median of 6,054 transcripts and 2262 genes per cluster (Fig. 2b and Supplementary Fig. S2c). Cell type determination was performed using marker genes curated from the literature (Supplementary Fig. S2c, Table S3). Similar to human HCC, mouse HCC tumors contained a preponderance (~30%) of myeloid cells, consisting of mostly macrophages and MDSCs, with a small subset of DCs (Supplementary Fig. S2d). The fraction of fibroblasts in the HCC tumors was similar to that in human HCC samples (2.7% of all cells). With the exception of cholangiocytes, all cell types were represented in all mouse HCC tumors (Supplementary Fig. S2d).

To further discern fibroblast heterogeneity, we then sorted 1746 CAFs in our scRNA-seq analyses derived from 7 murine HCC tumors and clustered the CAFs into 7 subpopulations (Fig. 2c). All 7 subclusters expressed high levels of canonical fibroblast markers, such as *Col1a2* and *Col1a1* (Fig. 2e and Supplementary Fig. S2c); however, each subcluster displayed distinct transcriptomic signatures (Fig. 2d, e and Supplementary Fig. S2e). Interestingly, we found that all 5 CAF subtypes identified in human HCC tumors were also represented in mouse HCC tissues (Fig. 2c). Furthermore, the 5 mouse CAF subsets possessed populations and gene signatures similar to those of human CAFs, including vCAFs, mCAFs, CD36^+^CAFs, lpCAFs and apCAFs (Supplementary Fig. S2e, f). The upregulated pathways, including ROS pathway and cholesterol metabolism were enriched in CD36^+^CAFs and lpCAFs (Fig. 2f and Supplementary Fig. S2g, h). Finally, we performed mIF staining using specific markers, including Myh11, Lum, Acta2, Cd36 and H2-Aa, to confirm that the main CAF clusters also existed in murine HCC tissues (Fig. 2g).

### Spatial heterogeneity of CAFs in murine and human HCC tissues

An interesting issue is the spatial distribution of CAF clusters in human and mouse HCC. To address this, we performed mIF assays and found that CD36^+^ CAFs showed more enrichment in the tumor core region than in the peritumor region in both human and mouse HCC tumors (Supplementary Fig. S3a, b), which was further validated in another HCC scRNA-seq database (GSE156625; Supplementary Fig. S3c, d)^17^ and through bulk RNA-seq^18,19^ using CIBERSORTx (Supplementary Fig. S3e). Additionally, we found that the abundances of lpmCAFs and lpCAFs were more enriched in BCLC-B than in BCLC-A stage HCC (Supplementary Fig. S3f). To explore the CAF subtype distribution in tumor and adjacent tissues, we isolated fibroblasts in human adjacent liver tissues using fluorescence-activated cell sorting (FACS) (Supplementary Fig. S4a) and found that the cells could be clustered into the same five CAF subtypes. However, we found that CD36^+^ CAFs were specifically enriched in HCC tumors (Supplementary Fig. S4b), while vCAFs showed a slightly significantly higher population in tumors than in adjacent liver tissues (Supplementary Fig. S4b, c). Finally, our results for the CAF subtypes were also clearly verified in the scRNA-seq data reported by Ma et al.^20^ (Supplementary Fig. S4d, e).

### Cell-state transition trajectory of different CAF subpopulations

To understand the underlying evolution of cellular status among CAF subtypes, we derived the pseudotime cell trajectory of the various CAF subtypes based on the Monocle 2 algorithm (Supplementary Fig. S4f, g). The inferred state transition trajectory contains two lineages, presenting a bifurcated structure from the progenitor state to the terminal effect state (Supplementary Fig. S4f, g). Both lineages start from the “progenitor” state and diverge after the intermediate state. Interestingly, by combining the findings from both the clustering and pseudotime analyses, we observed that mCAFs characterized by CAF transdifferentiation were located in the progenitor state. Then, lpmCAFs (CD36^+^ CAFs) were located in the intermediate state and finally diverged into vCAFs, lpCAFs and apCAFs, indicating a dynamic transition toward the terminal effect state, including vascular smooth muscle contraction, lipid metabolism and antigen-presenting processes characterized by *MUSTN1*, *APOC1* and *CD74* expression (Supplementary Fig. S4h). Similarly, in terms of murine CAF subpopulations, we found that mCAFs and proliferating CAFs were again located in the progenitor state; then, CD36^+^ CAFs and MeCAFs were located in the intermediate state and finally diverged into vCAFs, lpCAFs and apCAFs. Furthermore, the gene patterns involved in the CAF state transition were dissected (Supplementary Fig. S4h, i). The mCAFs with *LUM*, *DCN*, *VCAN* and *POSTN* expression were significantly reduced, whereas genes related to lpCAFs and apCAFs, including *APOC1*, *CD74*, and *HLA-DRA*, were significantly increased in both human and mouse CAFs (Supplementary Figs. S4h–j, k, l). Meanwhile, the transcription factors (TFs) related to lipid metabolism and antigen presentation processes, such as *CEBPA*, *MAFB*, and *IKZF1* (Supplementary Fig. S4i), were gradually upregulated along with the trajectory differentiation process. Conversely, some well-known factors, such as *CEBPB*, *NFIC*, *TWIST1* and *CREB3L1*, were downregulated over the course of the process (Supplementary Fig. S4i) and are involved in regulating CAF transdifferentiation and lipid metabolism.

### The interactions between different CAFs and MDSCs and the underlying mechanisms

To explore the interactions between HCC cells and niche cells, we conducted intercellular interaction analyses of 9 cell types based on ligand receptor pairs (Supplementary Fig. S5a, b). We detected 71 significant ligand-receptor pairs among the 9 cell groups, which were further categorized into 25 signaling pathways, including the TGFβ, noncanonical WNT (ncWNT), TNF, SPP1, PTN, PDGF, CXCL, CCL, and macrophage migratory inhibition factor (MIF) pathways (Supplementary Fig. S6a, b). Interestingly, cross-species analysis showed that MIF-CD74/CXCR4 was ranked top among interactions between CAFs and other cells, including B cells, DCs, MDSCs, monocytes or macrophages, NK cells and T cells (Supplementary Fig. S6b, c). The inferred MIF signaling network further revealed that tumor cells and CAFs are prominent sources of MIF ligands acting on MDSCs (CD33^+^CD11b^+^HLA-DR^lo^), monocytes or macrophages and DCs (Supplementary Fig. S6d). Additionally, we investigated the specific CAF subtypes that act as prominent sources of MIF ligands acting on MDSCs. The results showed that lpmCAFs and lpCAFs were the most prominent MIF ligand sources (Supplementary Fig. S6e–h). Finally, our cross-species analysis showed that *MIF* expression was mainly derived from CD36^+^ CAFs and lpCAFs (Supplementary Fig. S6i), which were prominent sources for MIF ligands acting on MDSCs and DCs (Supplementary Fig. S6i).

### CAF subtype-specific transcription factors and gene regulatory networks

**Fig. 3:**
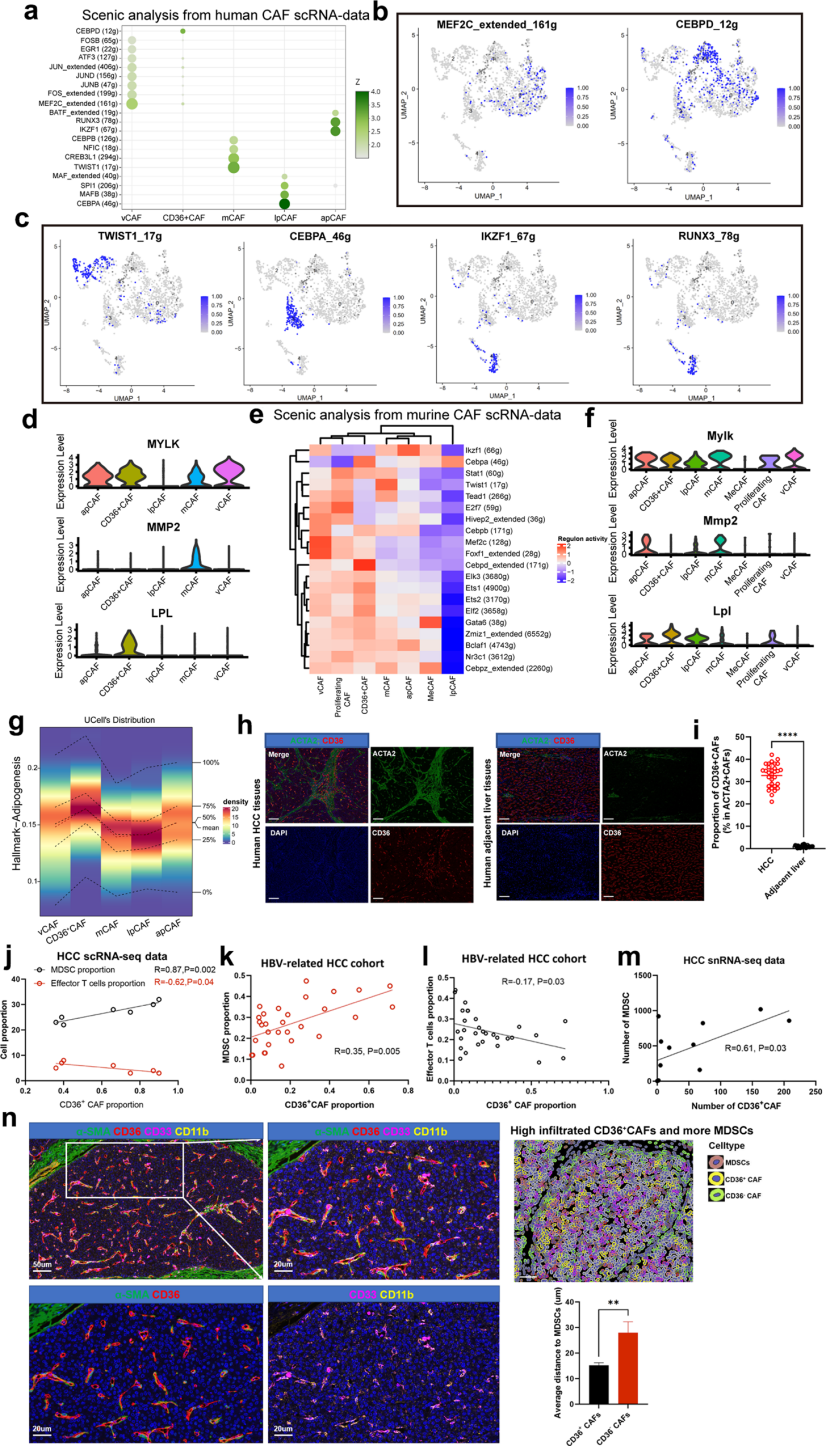
CAF subtype-specific TFs and the interactions between different CAFs and MDSCs. **a** Dotplot shows transcriptional factors enriched in different human CAF subclusters. **b**, **c** UMAP plots show top expressed TFs from CAF clusters. **d** Violin plots show targeted genes from TFs in human CAF clusters. **e** Heatmap show TFs enriched in different murine CAF subclusters. **f** Violin plots show targeted genes from TFs in murine CAF clusters. **g** The activity of adipogenesis pathway gene signatures in different human CAF subclusters. **h**, **i** The mIF show CD36^+^CAFs significantly enriched in human HCC than adjacent liver tissues. **j** The correlation between CD36^+^CAFs and MDSCs, effector CD8^+^T cells from scRNA-seq data. **k** The correlation between CD36^+^CAFs and MDSCs in HBV-related HCC cohort. **l** The correlation between CD36^+^CAFs and effector CD8^+^T cells in HBV-related HCC cohort. **m** The correlation between CD36^+^CAFs and MDSCs from snRNA-seq data. **n** MIF staining showed CD36^+^CAFs interacted with MDSCs in closer proximity. Data shown as mean ± S.E.M., one-way ANOVA following multiple comparison test was used, ***P < 0.001, **P < 0.01, *P < 0.05, and ns not significant.

We next sought to identify TFs and their targeted gene regulatory networks to better understand how CAF subtypes are established and maintained genetically. For this, we applied the software SCENIC^21^ to identify TFs and their targets that are highly active in one CAF subtype versus others. We observed that vCAFs were enriched in MEF2C and FOS (Fig. 3a, b), which was previously suggested to regulate neoangiogenesis^22^. In addition, target genes for MEF2C, such as *MYLK*, *ACTA2*, and *MYH11*, were upregulated in vCAFs (Fig. 3d). *TWIST1* and *CREB3L1* were among the TFs most highly expressed in mCAFs (Fig. 3a, c), and *TWIST1* is a key factor in CAF transdifferentiation^23^. Furthermore, target genes for TWIST1 (*COL1A1*, *MMP2*) were most upregulated in mCAFs (Fig. 3d and Supplementary Fig. S1g). lpmCAFs (CD36^+^ CAFs) showed high expression of *CEBPD* (Fig. 3a, c), a key regulator known to control transcriptional programs associated with lipid metabolism and EMT^24^. The target gene for lipoprotein lipase (LPL) was upregulated in lpmCAFs (Fig. 3d). In addition to *CEBPD*, lpCAFs showed high expression of *CEBPA* and *MAFB* (Fig. 3a, c), known TFs involved in promoting LDL metabolism transcriptional programs^25,26^. Finally, apCAFs showed high expression of *IKZF1* and *RUNX3* (Fig. 3a, c), which are implicated in macrophage polarization and T-cell recruitment^27,28^.

To detect master regulators that are active in these murine CAF subpopulations, SCENIC analysis was conducted and showed that *Mef2c* and *Foxf1* were active TF genes in murine vCAFs, similar to human vCAFs (Fig. 3e, f). Similarly, *Twist1*, *Cebpd*, *Cebpa* and *Ikzf1* were also active TFs in murine mCAFs, CD36^+^ CAFs, lpCAFs and apCAFs, respectively (Fig. 3e). Immunostaining assays further showed co-staining of these highly expressed TFs in different CAF subtypes in both human and murine HCC tumors (Supplementary Fig. S7a). Collectively, these results identify key TFs driving or maintaining the gene expression programs in identified CAF subtypes, providing further insights into the gene regulatory networks underlying CAF heterogeneity in HCC tumors.

### CD36^+^ CAFs are positively correlated with MDSCs and are in close proximity to MDSCs in HCC tumors

Following the identification of CAF subtypes in human or murine HCC tumors, we next explored the specific crosstalk mechanisms between CAFs and other cells in the TME. Notably, among all known ligand-receptor pairs, the MIF signaling pathway is dominated by MIF ligand and its CD74/CXCR4 receptor. Interestingly, lpmCAFs and lpCAFs, which commonly and highly expressed *CD36* (Fig. 1g), were the most prominent sources of MIF ligands acting on MDSCs (Supplementary Fig. S6g). Further IHC experiments showed that CD36 expression was mainly located in HCC stromal regions (Supplementary Fig. S7b). We therefore identified *CD36* in these two subclusters, lpmCAFs (c1-mCAF-CD36) and lpCAFs, using specific surface markers and found that they were both enriched in adipogenesis pathways (Fig. 3g) and accounted for ~35% of the CAF population in HCC, indicating their crucial role in HCC progression. Further mIF experiments showed that CD36^+^ CAFs infiltrated specifically in tumor tissues compared with adjacent liver tissues in both murine and human tissues (Fig. 3h, i and Supplementary Fig. S7c, d). Survival analysis showed that a high number of CD36^+^ CAFs indicates poor prognosis in HCC patients (Supplementary Fig. S7e).

First, we investigated whether there was a correlation between CD36^+^ CAFs and MDSCs, effector T cells within the TME. Our scRNA-seq data and TCGA data showed a positive correlation between CD36^+^ CAFs and MDSCs (Fig. 3j and Supplementary Fig. S7f), but an inverse correlation was observed for effector T cells (*GZMB*^+^ *CD8*^+^) (Fig. 3j), which was validated by IF assays in paraffin-embedded tumor sections from 30 primary HBV-related cases (Fig. 3k–l). Furthermore, 12 HCC tumors were subjected to single-cell nucleus sequencing (snRNA-seq), and the number of CD36^+^ CAFs was found to be positively associated with MDSCs (Fig. 3m and Supplementary Fig. S7g–j). We next verified the interactions between CD36^+^ CAFs and MDSCs. mIF staining revealed that CD36^+^ CAFs were in closer proximity to MDSCs than CD36^–^ CAFs (Fig. 3n), which was verified in *CTNNB1*^*N90*^;*Trp53*^*KO*^ HCC murine tissues (Supplementary Fig. S7k). Collectively, these results suggested that CD36^+^CAFs might confer potent immunosuppressive effects in HCC.

### Isolation of CAF subtypes from HCC tumors via flow cytometry

**Fig. 4:**
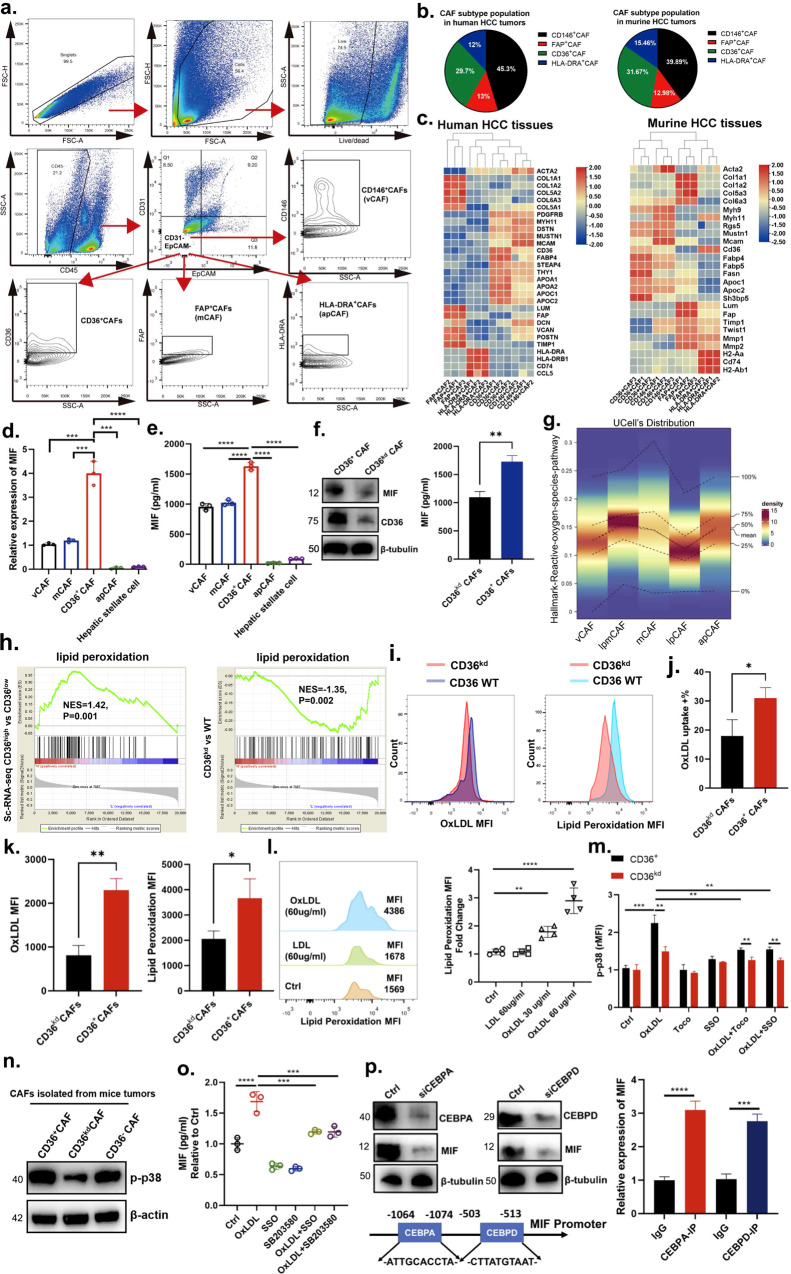
CD36 mediates OxLDL uptake to promote MIF expression via the lipid peroxidation/p38/CEBPs axis in CD36^+^ CAFs. **a** Isolation of CAF subtypes from HCC tumors via flow cytometry. **b** The average proportion of CAF subtypes in human or murine HCC tumors by flow cytometry. **c** Heatmap showed specific gene markers in CAF subtypes from murine and human HCC tumors. **d**, **e** The qPCR and ELISA assays showed MIF expression was higher in CD36^+^CAFs among all fibroblasts. **f** The ELISA and western blot assays showed MIF expression was downregulated when CD36 was knockdown in CAFs. **g** The activity of reactive oxygen species pathway gene signatures in different CAF subclusters. **h** GSEA shows top enriched pathways in CD36^high^ vs CD36^low^ group and CD36^kd^ vs WT group. **i**–**k** Uptake of OxLDL and lipid peroxidation in CD36^+^ or CD36^kd^ CAFs was measured using fluorescently conjugated OxLDL and flow cytometry. **l** Human CD36^+^CAFs treated with vehicle Ctrl, LDL (60 μg/mL), or OxLDL (30 or 60 μg/mL) for 24 h and then washed in PBS and incubated with BODIPY 581/591 C11 for the lipid peroxidation assay. **m** CD36^+^ or CD36^kd^ CAFs were treated with vehicle Ctrl, OxLDL (60 μg/mL), Toco (200 mM), SSO (100 mM), a combination of OxLDL (60 μg/mL) and Toco (200 mM), or a combination of OxLDL (60 mg/mL) and SSO (100 mM) for another 24 h. p38 phosphorylation (p-p38) was measured by flow cytometry, and the MFI of p-p38 was normalized to Ctrl. **n** The expression of p-p38 among CD36^+^CAFs, CD36^kd^CAFs and CD36^–^CAFs from in vivo HCC murine models. **o** CD36^+^CAFs were treated with vehicle Ctrl, OxLDL (60 μg/mL), SSO (100 mM), p38 inhibitor SB203580, a combination of OxLDL and SSO, or a combination of OxLDL and SB203580 for another 24 h. MIF secretion was measured by ELISA experiments, and the expression of *MIF* was nomalized to Ctrl. **p** CEBPA and CEBPD in CD36^+^CAFs modulated the transcriptional expression of *MIF* by ChIP assays. Data are mean ± s.d. of *n* = 3 independent experiments. *P < 0.05, **P < 0.01, ***P < 0.001 by Student’s *t* test. *n* = 3 biological replica*t*es. Data shown as mean ± S.E.M., one-way ANOVA following multiple comparison test was used, ***P < 0.001, **P < 0.01, *P < 0.05, and ns not significant.

To isolate and characterize CAF subpopulations, we examined our single-cell data and identified surface proteins that were uniquely expressed in each CAF subpopulation. In addition to CD36, which is unique to CD36^+^ CAFs and lpCAFs, we identified MHCII, MCAM and FAP as apCAF-, vCAF- and mCAF-specific surface markers, respectively (Supplementary Fig. S1g, h), and phenotypic function of these CAF subtypes identified by surface markers were validated by those of previous studies^12,29,30^. Following the exclusion of immune and epithelial cells, we gated on *ACTA2*-positive cells (Fig. 4a). Using CD36, CD146, FAP and MHCII antibodies, CAFs were segregated into four populations: (1) CD36-positive, (2) MHCII-positive, (3) FAP-positive, and (4) CD146-positive cells, presumably corresponding to CD36^+^ CAFs, apCAFs, mCAFs and vCAFs, respectively (Fig. 4a), and these results were also validated in HCC tissues using mIF assays (Supplementary Fig. S8a). Flow cytometry analysis showed an approximately similar proportion of CAF subtypes in both murine and human HCC tumors within the CD31 and EpCAM double-negative population (Fig. 4b). To validate this strategy for CAF sorting, cells were accordingly isolated via FACS, and qPCR analysis was performed. All four CAF subpopulations showed high expression of the pan-fibroblast markers *Col1a1*, *Col2a1*, and *Acta2* (Fig. 4c). CD36-positive CAFs were unique in their high expression of *Cd36*, *Fabp4*, and *Sh3bp5*, as well as the lpCAF markers *Fabp5*, *Apoc1* and *Fasn* (Fig. 4c). MHCII-positive CAFs were unique in their high expression of *Cd74* and *H2-Ab1* (Fig. 4c). FAP-positive cells showed high relative expression of the mCAF markers *Lum*, *Col5a1*, *Col6a3*, *Timp2*, *Mmp2* and *Twist1* (Fig. 4c). Mcam-positive CAFs showed high expression of the vCAF markers *Myh11*, *Myh9*, *Mustn1* and *Rgs5* (Fig. 4c). Finally, FAP-positive cells showed significantly higher relative expression of mCAF chemokines, such as *Lum*, *Mmp2*, and *Mmp1*, suggesting an enrichment of mCAFs in this population (Fig. 4c). Although CD36^+^ CAFs were identified as a subtype of fibroblasts in murine HCC tumors, they highly expressed genes that were also found in hepatic stellate cells (HSCs), such as Lrat and Gfap^31^, rather than expressing the mesothelial cell markers *Gpm6a* and *Msln* and the portal fibroblast markers Ds and *Entpd2*, raising the possibility that CD36^+^ CAFs originated from HSCs (Supplementary Fig. S8b, c). To test this hypothesis, we first performed transcriptomics to examine the gene signature of lipid metabolism commonly expressed in CD36^+^ CAFs and HSCs compared with CD36^–^ CAFs (Supplementary Fig. S8d). Then, we sought to establish the cellular origin of CD36^+^ CAFs in murine HCC tumors by using a lineage-tracing strategy. We selected transgenic mice to identify a putative HSC marker (*Lrat-Cre*; *Rosa26-LSL-tdTomato*; Supplementary Fig. S8e). In murine HCC tumors, approximately 96% of CD36^+^ACTA2^+^ fibroblasts were *Lrat* lineage-positive cells (Supplementary Fig. S8f, g). Together, these results suggest that *Lrat*-lineage stromal cells are a major source of CD36^+^ CAFs during murine HCC development.

### CD36 mediates OxLDL uptake to promote MIF expression via the lipid peroxidation/p38/CEBPs axis in CD36^+^ CAFs

To test the MIF levels produced by different CAFs, we isolated the different CAF subtypes via FACS. Further qPCR and ELISA experiments showed that the expression of *MIF* derived from CD36^+^ CAFs was significantly higher than that derived from vCAFs, mCAFs, apCAFs and HSCs (Fig. 4d, e). Based on these results, we thereafter focused on this subcluster of CD36^+^ CAFs for further study. We also isolated and expanded CD36^+^ CAFs from HCC tissues. Western blotting and ELISA analyses showed that CD36^+^ CAFs expressed and secreted less MIF when *CD36* was silenced in CD36^+^ CAFs (CD36^kd^ CAFs; Fig. 4f), indicating that *MIF* expression may be regulated by CD36. Importantly, these results suggest that CD36^+^ CAFs may facilitate the acquisition of certain tumor-promoting functions in MDSCs via paracrine signaling.

CD36 is a scavenger receptor that functions in lipid metabolism and has been reported to be involved in inflammatory responses and metabolic disorders, such as diabetes and obesity^32^. In the immune system, CD36 has been reported to mediate dendritic cell antigen acquisition and presentation^33^ and to support regulatory T-cell function^33^. However, little is known about its role in CAFs. Similar to the signaling pathways identified through our CAF scRNA-seq analyses (Fig. 4g), we found that CD36^+^ CAFs had higher expression of genes associated with lipid peroxidation than CD36^kd^ CAFs (Fig. 4h).

OxLDL is another abundant source of phosphocholine (OxPL) in tissues, which was higher in tumor interstitial fluid than in circulating systems (Supplementary Fig. S9a). Additionally, the level of OxLDL in anti-PD-1 non-response group was significantly higher than in response group by immunofluorescence assays (Supplementary Fig. S9b). Using fluorescently conjugated OxLDL and flow cytometry, we found that CD36^+^ CAFs had higher rates of OxLDL uptake than CD36^kd^ CAFs (Fig. 4i, j). CD36^+^ CAFs also displayed a significant increase in lipid peroxidation compared with CD36^kd^ CAFs based on a BODIPY 581/591 C11 lipid peroxidation assay (Fig. 4k). Furthermore, we wanted to determine whether OxLDL increases lipid peroxidation in CD36^+^ CAFs. First, we measured the effect of OxLDL on lipid peroxidation in CD36^+^ CAFs, and the results revealed that OxLDL, but not LDL, enhanced lipid peroxidation in CD36^+^ CAFs in a dose-dependent manner (Fig. 4l).

Oxidative stress, including lipid peroxidation, can activate p38 kinase and its downstream signaling pathways^34,35^. Therefore, we examined whether OxLDL can activate p38 by measuring p38 phosphorylation in CD36^+^ CAFs. We found that OxLDL induced p38 phosphorylation in CD36^+^ CAFs but to a much lesser extent in CD36^kd^ CAFs, and the addition of Toco or SSO diminished OxLDL-induced p38 phosphorylation (Fig. 4m). Furthermore, p38 phosphorylation showed higher enrichment in CD36^+^ CAFs than in CD36^kd^ or CD36-negative CAFs in vivo (Fig. 4n). This suggests that OxLDL promotes p38 activation through CD36 and lipid peroxidation. Next, we wanted to determine whether p38 acts downstream of OxLDL-CD36 signaling to promote *MIF* expression; thus, we treated CD36^+^ CAFs with OxLDL in the presence or absence of the p38 inhibitor SB203580 in vitro. The results revealed that p38 inhibition partially rescued the secretion of MIF in the presence of OxLDL (Fig. 4o), indicating that OxLDL promotes MIF secretion in part through p38 activation.

From our SCENIC analysis, we found that CEBP family (CEBPA and CEBPD) TFs, reported to be crucial for lipid metabolism, were enriched specifically in CD36^+^ CAFs (including lpmCAFs and lpCAFs). Previous studies have identified p38MAPK as a key regulator of CEBPA and CEBPD activation^25,36^. Therefore, we first examined whether MIF secretion is dependent on p38-induced CEBPA and CEBPD activation in CD36^+^ CAFs. When *CEBPA* and *CEBPD* were silenced separately, we observed that MIF was significantly decreased in CD36^+^ CAFs treated with OxLDL (Fig. 4p). Then, we performed bioinformatics analysis to identify the potential binding sites of CEBPA or CEBPD in the *MIF* promoter (Fig. 4p). Next, Chromatin Immunoprecipitation (ChIP) assays were conducted and revealed that both CEBPA and CEBPD could directly bind to the MIF promoter (Fig. 4p). Collectively, these results demonstrate that CD36 mediates OxLDL uptake to promote *MIF* expression via the lipid peroxidation/p38/CEBP axis.

### CD36^+^ CAF-derived MIF potentiates the capacity of MDSCs to promote an immunosuppressive TME and tumor stemness via IL-6/STAT3 activation

Based on the above results indicating that the population of CAFs is associated with the number of MDSCs, we hypothesized that CD36^+^ CAF-derived MIF promotes CD33^+^ MDSC expansion. Recent studies have shown that CD33^+^CD11b^+^HLA-DR^–/lo^ cells from cancer patients have a complete overlap with monocytic-MDSCs and PMN-MDSCs (Supplementary Fig. S9c), which were often defined as cells co-expressing CD14 and CD15^37,38^. This did not allow us to isolate different types of MDSCs for further study. As reported, MIF can induce monocytic MDSC accumulation within tumors^39,40^. Furthermore, monocytic MDSCs have been thought to be the more suppressive because this population is able to suppress both antigen-specific and nonspecific T-cell proliferation^39^, which promotes tumor growth and metastasis.

**Fig. 5:**
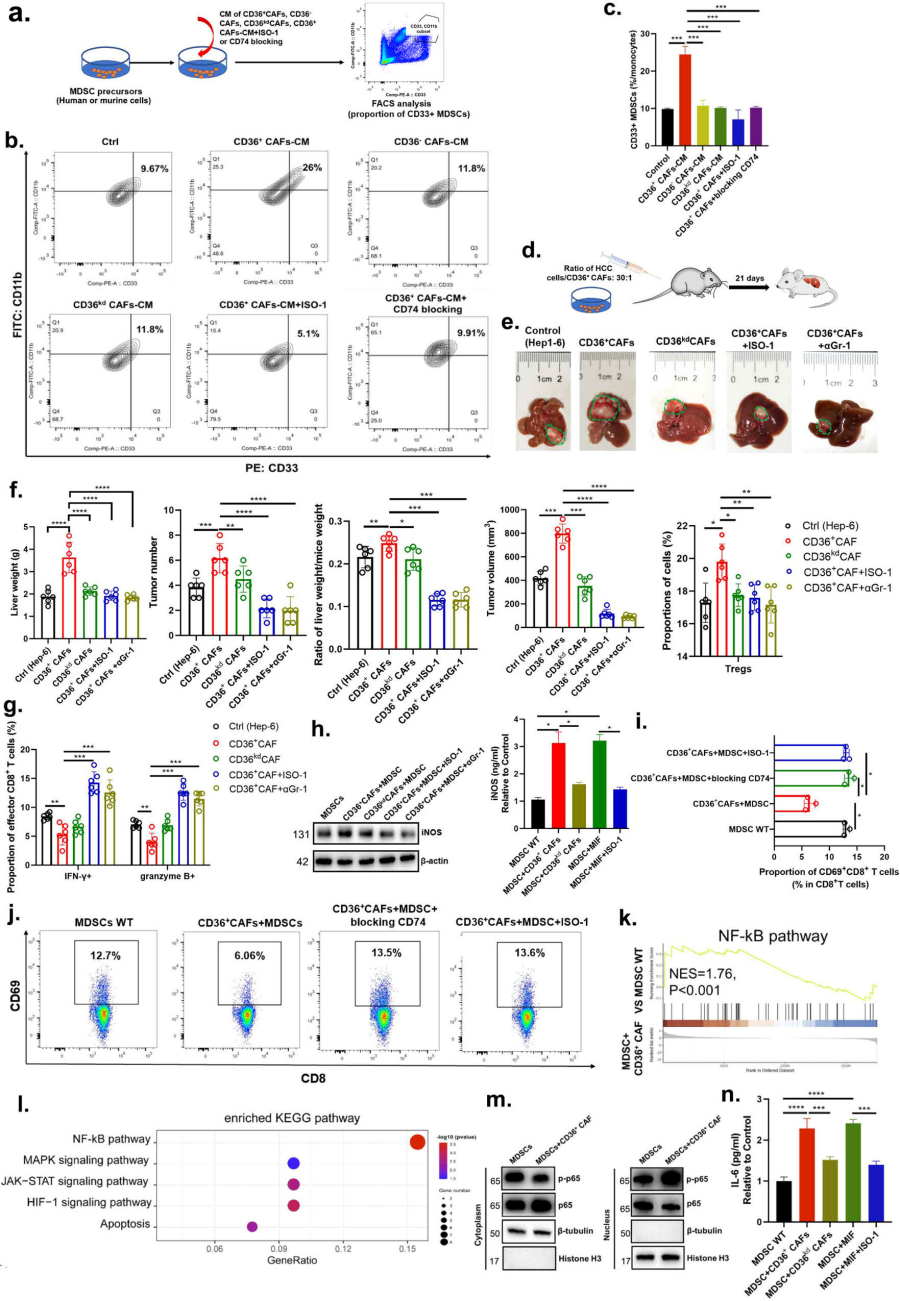
CD36^+^ CAF-derived MIF potentiates the capacity of MDSCs to promote an immunosuppressive TME and tumor stemness via IL-6/STAT3 activation. **a** The schematic diagram showed co-culture of MDSCs precursors with condition media (CM) of CD36^+^CAFs, CD36^–^CAFs, CD36^kd^CAFs, CD36^+^CAFs+ISO-1 or blocking CD74. **b**, **c** MDSC proportion was measured by flow cytometry when co-cultured with vehicle Ctrl, CD36^+^ CAFs, CD36^–^ CAFs, CD36^kd^ CAFs, a combination of CD36^+^ CAFs and MIF inhibitor ISO-1, or a combination of CD36^+^ CAFs and CD74 blocking agents. **d** The schematic diagram showed co-injection of tumor cells and CD36^+^CAFs at the ratio of 30:1 in orthotopic HCC model. **e** Representative images of HCC tumors from the orthotopic HCC model. **f** Liver weight, tumor numbers, tumor volume and ratio of liver weight and mice weight from the orthotopic HCC model. CAFs transduced with the empty lentiviral vector as a control. **g** The proportion of effector CD8^+^ T cells from different groups in the orthotopic HCC model. **h** The western blot and ELISA assays showed iNOS was evaluated in MDSCs-WT, CD36^+^CAFs+MDSCs, a combination of CD36^+^CAFs+MDSCs and ISO-1 or αGr-1. **i**, **j** The proportion of CD69^+^CD8^+^ T cells was evaluated in MDSCs-WT, CD36^+^CAFs+MDSCs, a combination of CD36^+^CAFs+MDSCs and ISO-1 or blocking CD74 by flow cytometry. **k**, **l** GSEA and KEGG analysis shows top pathways enriched in MDSC-CD36^+^CAF-CM vs MDSC-WT. **m** Western blotting experiment shows NF-kB pathway changes in MDSCs treating with CD36^+^ CAFs. **n** ELISA assay shows IL-6 secretion in MDSCs treating with CD36^+^ CAFs, CD36^kd^ CAFs, MIF or a combination of MIF and ISO-1. Data shown as mean ± S.E.M., one-way ANOVA following multiple comparison test was used, ***P < 0.001, **P < 0.01, *P < 0.05, and ns not significant.

First, to determine whether MIF from tumor cells or CD36^+^ CAFs mediates the regulation of CD33^+^ MDSC expansion, human or murine CD36^+^ CAFs were isolated from primary human HBV-related or murine HCC tumors. The conditioned medium (CM) of CD36^+^ CAFs (CD36^+^ CAFs-CM), CD36^–^ CAFs, CD36^kd^ CAFs (CD36^kd^ CAFs-CM), CD36^+^ CAFs+MIF inhibition (ISO-1) or CD74 blockade were used to treat MDSC precursors, and the proportion of CD33^+^ MDSCs was measured (Fig. 5a, b). CD36^+^ CAF-CM, MIF were found to increase the number of CD33^+^ MDSCs (Fig. 5c and Supplementary Fig. S9d, e). Similar to CD36^kd^ CAF-CM treatment, CD36^+^ CAF-CM + ISO-1 and CD36^+^CAF-CM + CD74 blockade showed good activity in suppressing CD33^+^ MDSC expansion (Fig. 5c and Supplementary Fig. S9d, e), suggesting that MIF secretion is a critical mediator of the regulation of MDSC expansion by CD36^+^ CAFs.

Then, using an orthotopic HCC model in immunocompetent mice, we demonstrated that coinjection of CD36^+^ CAFs (~30:1) isolated from murine HCC tumors significantly promoted tumor growth or metastasis of HCC tumor cells in the livers of mice, which was greatly blunted by specific knockdown of CD36, inhibition of MIF or depletion of Gr-1^+^ MDSCs (Fig. 5d, e). Furthermore, coinjection of CD36^+^ CAFs significantly increased the infiltration of Tregs but decreased effector CD8^+^ T cells in tumors (Fig. 5f, g), which was also greatly blunted by specific knockdown of CD36, inhibition of MIF or depletion of Gr-1^+^ MDSCs (Fig. 5f, g). These data indicated that the tumor-promoting effect of CD36^+^ CAFs was indirectly mediated by the immunosuppressive function of MDSCs.

MDSCs can promote immune suppression through several contact-independent mechanisms, including the expression of inducible nitric oxide synthase (iNOS), which is known to inhibit the cytotoxicity and function of CD8^+^ T and NK cells in solid tumors^41–43^. First, to investigate whether CD36^+^ CAFs can educate MDSCs to potentiate their immunosuppressive capacity via iNOS signaling, CM was collected from cultures of primary CD36^+^ CAFs, CD33^+^ MDSCs, or CD33^+^ MDSCs pretreated with CM from CD36^+^ CAFs, CD36^kd^ CAFs, or cells with CD74 blockade, and the level of iNOS was evaluated. CD36^+^ CAF-MDSC-CM, but not CD36^kd^ CAF-MDSC-CM or CM from cells with CD74 or MDSC blockade, drastically promoted iNOS secretion in MDSCs (Fig. 5h). Furthermore, our coculture assays showed that the proportion of CD69^+^, IFNG^+^ CD8^+^ T cells was significantly downregulated, but the proportion of Tregs and PD1^+^CD8^+^ T cells was upregulated in the CD36^+^ CAF + MDSC group compared with the MDSC-WT group, while CD36^+^ CAFs + MDSC + ISO-1 or CD74 blockade caused mild effects (Fig. 5i, j and Supplementary Fig. S9f), indicating that CD36^+^ CAFs potentiate the immunosuppressive capacity of CD33^+^ MDSCs in a fibroblastic CD36-dependent manner via the MIF/CD74/iNOS axis.

Additionally, we used an orthotopic HCC model in nude mice to demonstrate that coinjection of CD36^+^ CAFs significantly promotes tumor growth or metastasis of the tumor cells in the livers of nude mice, which was greatly blunted by specific knockdown of CD36, inhibition of MIF or depletion of Gr-1^+^ MDSCs (Supplementary Fig. S9g, h). Furthermore, coinjection of CD36^+^ CAFs significantly increased the frequencies of ALDH^+^ cells in tumors (Supplementary Fig. S9i), which was also greatly blunted by specific knockdown of CD36, inhibition of MIF or depletion of Gr-1^+^ MDSCs (Supplementary Fig. S9i). These data indicate that the tumor-promoting effect of CD36^+^ CAFs is indirectly mediated by the non-immunosuppressive function of MDSCs, likely the stemness-enhancing capacity.

To investigate whether CD36^+^ CAFs can educate MDSCs to potentiate their stemness-enhancing capacity via paracrine signaling, tumorsphere assays were performed and showed that CD36^+^ CAF-MDSC-CM, but not CD36^kd^ CAFs-MDSC-CM, drastically promoted tumorsphere formation efficiencies in HCC cell lines, whereas stimulation with CM from CD36^+^ CAFs or CD33^+^ MDSCs caused only mild effects (Supplementary Fig. S9j, k). Similar patterns were observed in stemness marker gene expression (Supplementary Fig. S9l). CM from CD33^+^ MDSCs pretreated with CD36^+^ CAF CM, but with MIF inhibition or CD74 blockade lacked such effects (Supplementary Fig. S9l). Collectively, these results suggest that CD36^+^ CAFs potentiate the stemness-enhancing capacity of CD33^+^ MDSCs in a fibroblastic CD36-dependent manner via the paracrine MIF-CD74 axis.

Finally, to explore the molecular mechanisms by which CD36^+^ CAF-derived MIF affect MDSCs, we conducted RNA-seq and compared the MDSC-MIF and MDSC groups. The results showed that the nuclear factor kappa B (NF-κB) signaling pathway, which was ranked as the top pathway and has been reported to regulate cytokine secretion in MDSCs, was activated in MDSCs pretreated with CD36^+^ CAF-derived MIF (Fig. 5k, l). To investigate the downstream signals underlying the mechanical stimuli leading to CD36^+^ CAF-associated MDSC expansion, we first examined the phosphorylation of NF-κB protein p65 in MDSCs induced by CD36^+^ CAFs and in those from the peripheral blood of HCC patients. We found that the phosphorylation of p65 was increased in MDSCs induced by CD36^+^ CAFs (Fig. 5m). p65 activation has been reported to significantly induce iNOS and IL-6 secretion^44,45^. ELISA results showed that iNOS and IL-6 secretion was significantly upregulated in MDSCs induced by CD36^+^ CAFs or MIF compared with controls (Fig. 5n).

Furthermore, previous studies have shown that monocytic MDSCs can induce cancer stemness through IL-6/STAT3 activation^44^, which is important for the growth and metastasis of tumor cells. Thus, we investigated whether STAT3 activation is involved in MDSC-stimulated HCC stemness. To test this hypothesis, we blocked STAT3 signaling in MHCC97H cells cocultured with MDSCs pretreated with CD36^+^ CAFs or CD36^kd^ CAFs. The STAT3 inhibitor Stattic significantly reduced sphere formation of cancer stem cells (CSCs) and the expression of OCT4 and SOX2 induced by MDSCs (Supplementary Fig. S9m). Collectively, these results suggest that MDSCs induced by CD36^+^ CAFs may promote CSCs via IL-6-mediated STAT3 activation in HCC.

### Targeting CD36 synergizes with immunotherapy in HCC murine models

**Fig. 6:**
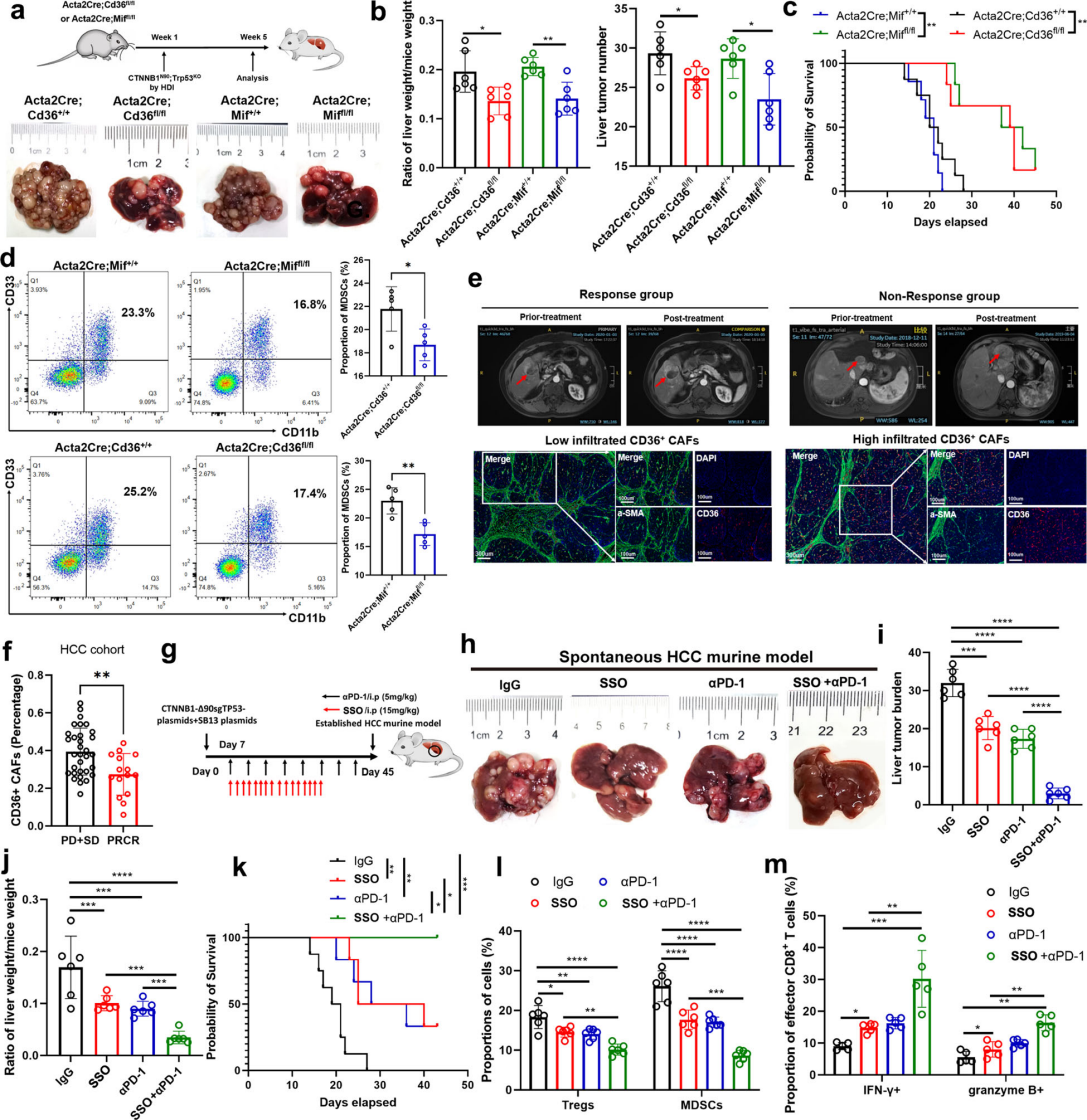
CD36^+^ CAFs predict efficacy of HCC immunotherapy and targeting MIF synergizes with immunotherapy in HCC murine model. **a**–**c** The HCC initiation and progression were evaluated in *Cd36* and *Mif* conditional knockout (*Acta2Cre*) mice. **d** The proportion of MDSCs was downregulated in *Cd36* and *Mif* conditional knockout (*Acta2Cre*) mice. **e**, **f** The prediction performance of CD36^+^ CAFs in HCC immunotherapy. **g**–**k** CD36 inhibitor SSO sythesizes PD-1 blockade in C57/BJ6 spontaneous HCC model. **l**, **m** The changes of Tregs, MDSC, IFN-γ^+^, GZMB^+^ CD8^+^ T cells in four different groups. Data shown as mean ± S.E.M., one-way ANOVA following multiple comparison test was used, ***P < 0.001, **P < 0.01, *P < 0.05, and ns not significant.

According to the above findings, CD36^+^ CAF-derived MIF promoted immunosuppressive MDSC accumulation and accelerated HCC progression. To further investigate whether CD36^+^ and MIF^+^ CAFs play a role in HCC initiation, we established *Cd36* (*Acta2*^*Cre*^;*Cd36*^*fl/fl*^) and *MIF* (*Acta2Cre;Mif*^*fl/fl*^) conditional knockout 6-week-old mice to determine that HCC tumor burden and the proportion of MDSCs were significantly reduced when *Cd36* or *MIF* was knockout in vivo (Fig. 6a–d), which indicated CD36^+^ CAFs were involved in HCC initiation. Monocytic MDSCs have been reported to be immunosuppressive and correlated with poor response to immunotherapy in cancer^46^. Additionally, the cancer stemness induced by MDSCs has been found to promote immunotherapy resistance in multiple cancer types^37,38^. Therefore, therapeutically targeting or reducing MDSCs combined with immune checkpoint blockade agents could enhance T-cell immunotherapy and achieve optimal antitumor efficacy. To this end, we also hypothesized that the population of CD36^+^ CAFs was associated with the efficacy of immunotherapy in HCC patients.

Hence, we first investigated CD36^+^ CAFs in our cohort of resected HCC tissues from patients administered neoadjuvant anti-PD-1 therapy (toripalimab in combination with lenvatinib as neoadjuvant therapy for resectable hepatocellular carcinoma; clinical trial number: NCT03867370). The results showed that CD36 and α-SMA coexpression was lower in the anti-PD-1 response group than in the nonresponse group, indicating that a low number of CD36^+^ CAFs is predictive of a better HCC immunotherapy response (Fig. 6e, f). Then, we explored the efficacy of single agents (CD36 inhibitor or anti-PD-1 therapy) and combination treatment strategies (CD36 inhibition and anti-PD-1 therapy) in a C57/BJ6 *CTNNB1*^*N90*^;*Trp53*^*KO*^ HCC model and our established anti-PD1 resistant HCC. Interestingly, the combination therapy exhibited marked antitumor efficacy in these HCC murine models (Fig. 6g–k and Supplementary Fig. S10a–h) and altered the immune landscape toward antitumor immunity, with decreased proportions of Tregs and MDSCs and increased proportions of IFN-γ^+^ and granzyme B^+^ CD8^+^ T cells (Fig. 6l–m). Thus, a low number of CD36^+^ CAFs in tumors might predict a better immunotherapy response in HCC, and targeting CD36 with SSO can be used to synergistically enhance the efficacy of immunotherapy.

## Discussion

In this study, we employed scRNA-seq to comprehensively delineate the transcriptomic landscape of human HCCs and revealed novel cellular interactions between HCC cells, MDSCs and CD36^+^ CAFs at single-cell resolution. HBV-related HCC tissues are characterized by intense liver fibrosis and desmoplastic reactions, during which activated CAFs surrounding HCC cells are believed to play a pivotal role in HCC progression. However, the cellular diversity of CAFs and how CAF subsets interact with HCC cells or other immune cells have not been well defined at single-cell resolution.

scRNA-seq analysis has been used to elucidate constituent cell types, including CAFs, in multiple cancer types^5–8,11,12^. We identified 5 common and distinct CAF subsets via cross-species analysis, namely, vCAFs, mCAFs, lpmCAFs, lpCAFs and apCAFs, in HCC tissues. Previous studies have shown that CAFs and HCC cells engage in crosstalks mediated through numerous molecular mechanisms^2,4,47^. However, these studies cannot exclude other mechanisms through which CAF subsets regulate HCC progression. By detailing the different subpopulations of CAFs present in HCC tumors, we found a marker gene (*CD36*) in both human and murine CAF subtypes, which were specifically enriched in HCC. SCENIC analysis further indicated that CAF subclusters take on diverse functions in vivo, while lipid metabolism pathways and CEBPs were highly enriched in both lpmCAFs and lpCAFs. Our study identified *CD36* as a marker of the lmpCAF and lpCAF populations. As a membrane glycoprotein, CD36 has been studied in many mammalian cell types, such as adipocytes, macrophages, and hepatocytes^32^. However, little is known about its role in CAFs. We then found that these CAFs secreted high levels of MIF compared with other CAFs, which was previously reported to promote MDSC accumulation in the TME^39,40^. Our data further showed that CD36^+^ CAF-derived MIF promotes immunosuppressive TME and cancer stemness by enhancing MDSC expansion and suppressing T-cell-mediated antitumor immunity.

Identifying factors that cause immune suppression in the TME can lead to the development of novel immunotherapies. Although oxidized lipids are a common feature of inflamed tissue^32,48^, the role of oxidized lipids in the TME has not yet been well addressed. Our study suggests a new mode of immunosuppression meditated by CAFs in the TME: increased import of oxidized lipids by CD36^+^ CAFs, likely caused by elevated lipid oxidation in tumors, leading to greater lipid peroxidation, activation of p38 kinase, and overexpression of *MIF* in CD36^+^ CAFs. Our study illuminates the immunomodulatory effects of oxidized lipids in HCC. Deregulated lipid metabolism is a hallmark of the TME, and a recent study showed that increased lipid uptake and accumulation are observed in many types of intratumoral immune cells and are often associated with impaired antitumor immune function. Along these lines, we found that Ox-LDL induced lipid peroxidation, p38 phosphorylation, and CEBPA/D activation in CD36^+^ CAFs and finally promoted MIF secretion in a CD36-dependent manner. Hence, our data expanded the findings of previous studies by identifying increased lipid uptake and accumulation induced by CAFs that promoted an immunosuppressive TME, thereby increasing HCC malignancy.

Our study reveals a mode of immunosuppression in the TME, opening an unappreciated link between lipid oxidation and cancer immunotherapy for further exploration. MIF can enhance the immunosuppressive TME by increasing the abundance of monocytic MDSCs within tumors. Our study further confirmed that targeting CD36 in the TME with SSO may serve as a therapeutic adjuvant to immunotherapy. The limitation of this study is the relatively low number of CAFs sequenced from HCC tissues. However, it is important to emphasize that our CAF subclusters can also be validated in other HCC scRNA-seq datasets.

Collectively, our findings provide a comprehensive HCC transcriptomic landscape at the single-cell level and identify novel mechanisms by which CD36^+^ CAF-secreted MIF induced by lipid peroxidation regulates the immune evasion of tumor cells. Targeting CD36 could effectively enhance the treatment efficacy of immunotherapy. Further investigation is required to determine the intratumoral signals that induce different CAF subtype formation and activation and to define the role that other CAF subtypes play in the TME and tumor immunity.

## Materials and methods

### Human HCC samples

Unstained paraffin-embedded 5 mm tissue sections of 30 HBV-related HCC, 12 frozen HCC tumor tissues, 7 fresh HBV-related and 7 non-HBV related HCC samples were obtained from the Zhongshan hospital of Fudan University (Shanghai, China), with Institutional Review Board approval (B2021-611) and all patients were consent to participate in this study.

### Cell lines

MHCC97H and Hep1-6 were human and mouse primary HCC cell lines, respectively. Briefly, primary HBV-related HCC tissues were minced with a scalpel in a tissue culture dish, then enzymatically dissociated in 10 mL of PBS with 0.1% collagenase I (Sigma-Aldrich, St. Louis, MO, USA) at 37 °C for 1 h with gentle agitation. The suspension was neutralized with complete medium and centrifuged at 300× *g* for 5 min. The cell pellet was suspended in alpha modification of Minimum Essential Medium (α-MEM) containing 10% selected fetal bovine serum (FBS) and 1 ng/mL basic fibroblast growth factor (bFGF, R&D Systems, Minneapolis, MN), 100 μg/mL penicillin, and 100 U/mL streptomycin (Invitrogen, NY, USA). The cells were grown in the culture dishes. After 48 h, non-adherent cells and tissue debris were removed and washed twice with PBS. Adherent fibroblasts were further incubated for 6-10 days until 80%–90% confluence. Then the mixed fibroblasts were labeled by CD36-APC, FAP, MCAM, HLA-DRA-PE and sorted by FACS to enrich CD36, FAP, MCAM, HLA-DRA positive CAFs. The corresponding CAFs were further expanded in the above medium (α-MEM + 10% selected FBS + 1 ng/mL bFGF) and passage 5–10 CAFs were used in this study.

### Animals

For HDTVi, vectors for HDTVi were prepared using the EndoFree-Maxi Kit (Qiagen) and resuspended in a sterile 0.9% NaCl solution/plasmid mix containing 10 μg of pX330-p53 (Addgene 59910) or pT3-N90-beta-catenin (Addgene 31785), and 10 μg of CMV-SB13 Transposase. CRISPR-Cas9 vector system carrying sgRNAs targeting *Trp53* together with the Sleeping Beauty Transposon system overexpressing *CTNNB1-N90* vector in sterile saline constituted a total volume of 10% of the mouse body weight were injected into the lateral tail vein of 6-week-old C57BL/6 J mice in 6–8 s^15,16^. HDTVi-induced tumors were harvested 3 weeks after HDTVi. For staining and lineage tracing assays, 3–6 mice (male and female) were used per group. 6-week-old mice male and female mice were purchased from The Jackson Laboratory, including C57BL/6J mice (JAX stock, #000664), *Acta2-iCre-PolyA* (Gem Pharmatech, #T036743), *Cd36em1*(*flox*) (Shanghai Model Organisms Center, #NM-CKO-200086), *Mifem1*(*flox*) (Shanghai Model Organisms Center, #NM-CKO-2110274), *Lratem1*(*2A-Cre*) (Shanghai Model Organisms Center, #NM-KI-190097), *R26-CAG-LSL-tdTomato* (Shanghai Model Organisms Center, # NM-KI-225042). All animals were housed in a pathogen-free facility with 24-h access to food and water. Animal experiments in this study were approved by and performed in accordance with the institutional animal care and use committee at the Zhongshan hospital, Fudan University. Mice were euthanized by cervical dislocation under anesthesia.

### Library preparation and sequencing

Single-cell RNA-seq libraries were prepared using the Chromium Single Cell 3ʹ Reagent Kits v3 (10× Genomics), according to the manufacturer’s instructions. Briefly, approximately 5000 cells/ FACS-sorted cells were washed with 0.04% BSA DPBS for three times and were resuscitated to a concentration of 700–1200 cells/μL (viability ≥ 85%). Cells were captured in droplets at a targeted cell recovery of cells. After the reverse transcription step, emulsions were broken and Barcoded-cDNA was purified with Dynabeads, followed by PCR amplification. Amplified cDNA was then used for 3ʹ gene expression library construction. For gene expression library construction, 50 ng of amplified cDNA was fragmented and end-repaired, double-size selected with SPRIselect beads, and sequenced on a NovaSeq platform (Illumia) to generate 150 bp paired-end Reads.

### Cell counting & quality control

The cells were washed with 0.04% BSA DPBS for three times and were resuscitated to a concentration of 700–1200 cells/μL (viability ≥85%) as determined using the Countess^®^ II Automated Cell Counter.

### Gel Beads-in-emulsion (GEM) geration & barcoding

GEMs are generated by combining Barcoded Single Cell 3ʹ v3 Gel Beads, a Master Mix containing cells, and Partitioning Oil onto Chromium Chip B. To achieve single cell resolution, cells are delivered at a limiting dilution, such that the majority (~90%–99%) of generated GEMs contain no cell, while the remainder largely contain a single cell. Immediately following GEM generation, the Gel Bead is dissolved, primers are released, and any co-partitioned cell is lysed. Primer that contains an Illumina TruSeq Read 1 (Read 1 sequencing primer), 16 nt 10× Barcode, 12 nt unique molecular identifier (UMI) and 30 nt poly(dT) sequence are mixed with the cell lysate and a Master Mix containing reverse transcription (RT) reagents. Incubation of the GEMs produces Barcoded, fulllength cDNA from poly-adenylated mRNA.

### Post GEM-RT cleanup & cDNA amplification

After incubation, GEMs are broken and pooled fractions are recovered. Silane magnetic beads are used to purify the first-strand cDNA from the post GEM-RT reaction mixture, which includes leftover biochemical reagents and primers. Barcoded, full-length cDNA is amplified via PCR to generate sufficient mass for library construction.

### Gene expression library construction

Enzymatic fragmentation and size selection are used to optimize the cDNA amplicon size. TruSeq Read 1 (Read 1 primer sequence) is added to the molecules during GEM incubation. P5, P7, a sample index, and TruSeq Read 2 (Read 2 primer sequence) are added via End Repair, A-tailing, Adaptor Ligation, and PCR. The final libraries contain the P5 and P7 primers used in Illumina bridge amplification. Qubit instrument was used to quantify the libraries, Agilent 2100 Bioanalyzer or 5300 Fragment Analyzer were applied to proceed quality control, and the final library size was about 450 bp.

### Sequencing and quality control

The libraries were sequenced on the NovaSeq platform (Illumia) to generate 150 bp paired-end Reads, according to the manufacturer’s instructions. Raw data (Raw Reads) of fastq files were assembled from the Raw BCL files using Illumina’s bcl2fastq converter. Raw data firstly were processed through primary quality control. The monitored quality assessment parameters were, (i) contain N more than 3; (ii) the proportion of base with quality value below 5 is more than 20%; (iii) adapter sequence. All the downstream analyses were based on the clean data with high quality.

### Generation and analysis of single-cell transcriptomes

Raw reads were demultiplexed and mapped to the reference genome by 10× Genomics Cell Ranger pipeline (https://support.10xgenomics.com/single-cell-geneexpression/software/pipelines/latest/what-is-cell-ranger) using default parameters. All downstream single-cell analyses were performed using Cell Ranger and Seurat^10,49^ unless mentioned specifically. In brief, for each gene and each cell Barcode (filtered by CellRanger), unique molecule identifiers were counted to construct digital expression matrices. Secondary filtration by Seurat: A gene with expression in more than 3 cells was considered as expressed, and each cell was required to have at least 200 expressed genes. After filtering out some of the foreign cells, cellranger count takes FASTQ files performs alignment, filtering, Barcode counting, and UMI counting. It uses the Chromium cellular Barcodes to generate feature Barcode matrices by cellranger count or cellranger aggr and reruns the dimensionality reduction, clustering, and gene expression algorithms using cellranger default parameter settings. Then, we use Seurat to perform secondary analysis of gene expression. Specifically, the Seurat package was used to normalize data, dimensionality reduction, clustering, differential expression. We used Seurat alignment method canonical correlation analysis (CCA)^9^ for integrated analysis of datasets. For clustering, highly variable genes were selected and the principal components based on those genes were used to build a graph, which was segmented with a resolution of 0.6.

### Trajectory inference for tumor-infiltrating immune cell subpopulations

The status of the tumor-infiltrating immune cell subpopulations in the TME is dynamic and they may differentiate into different cellular states that exert different biological functions, e.g., cancer fighting or cancer tolerant. We performed the trajectory analysis using pseudo-time inferencing algorithm Monocle 2^10,50^ to reconstruct the cell differentiation trajectory of different tumor-infiltrating immune cells. It uses a machine-learning technique called reversed graph embedding to describe multiple fate decisions in a fully unsupervised manner and derives a principal tree on a population of single cells that reveals the progression of cell and reconstruct their trajectory as a cell progresses through the biological process under study. Different branches in the cell trajectory likely distinguished molecularly distinct cell subpopulations (denoted by different cellular states) within a certain cell type.

### GO and KEGG pathway enrichment

GO and KEGG enrichment analysis of differentially expressed gene sets were implemented in the GOseq R and KOBAS 3.0 package, respectively. GO terms with adjusted *P*-value below 0.05 were considered as significantly enriched by DEGs.

### Spatial distribution of CAF subtypes analysis

The gene expression matrix of the integrated CAF scRNA-seq data, with the pan-CAF clustering labels, was uploaded to the CIBERSORTx^18^ web server to generate a gene expression profile (GEP), using default settings except that the numbers of minimum and maximum genes for the GEP were set to 50 and 150, respectively. With this GEP and default parameters, CIBERSORTx estimated the pan-CAF abundances in bulk RNA-seq samples that were derived from three to six regional biopsies of tumors from patients (one tumor per patient) with HBV-related HCC^19^. This RNA-seq data were downloaded from the GEO^19^.

### TF analysis

Using the TF database described by Lambert and colleagues^51^, we identified which of the CAF subtype marker genes were TFs. To identify gene regulatory networks, target genes of pan-CAF–specific TFs were identified and extracted from SCENIC (v1.1.2.2)^21^. We then evaluated whether the target genes of the identified TFs were enriched in the same pan-CAF subtypes.

### Cell-cell interaction analysis using CellChat

To enable a systematic analysis of cell-cell communication, we re-clustered distinct each cell type. The CellChat package (http://www.cellchat.org/) was adopted to explore the ligand-receptor pairs between niche cell subtypes and MDSC, malignant cells as previously reported^52^. We chose the receptors and ligands expressed in more than 10% of the cells in the specific cluster for subsequent analysis. The interaction between distinct cell subpopulations via putative ligand-receptor pairs was visualized using ggplot2 package.

### Cell type determination

Highly variable genes were identified as having a normalized expression between 0.125 and 3 as well as a quantile-normalized variance exceeding 0.5. Thereafter, principal component analysis was used to reduce the number of dimensions representing each cell. We adopted the first 20 principal components to further conduct tSNE or UMAP dimensionality reduction using the default settings of the Run tSNE and UMAP function. Cell types in the resulting two-dimensional representation were annotated to known biological cell types using canonical marker genes and the putative CNV signal.

### Distinguish malignant and non-malignant epithelial cells based on inferred CNVs

Initial CNVs for each region were estimated by inferCNV R package^53^. The CNVs of total cell types were calculated by expression level from scRNA-seq data for each cell with -cutoff 1 and -noise_filter 0.2. For each sample, gene expression of cell was re-standardized and values were limited as −1 to 1. The CNV score of each cell was calculated as quadratic sum of CNV region. Putative malignant cells were defined as those with CNV signal above 0.04 and non-malignant cells are defined as CNV signal below 0.04.

### Short hairpin RNA (shRNA) and lentivirus package

To generate the lentivirus plasmid for stable RNA interference, short hairpins were designed using online software (http://rnaidesigner.lifetechnologies.com/rnaiexpress/design.do). The sequence of the effective shRNAs were provided as follows: shCD36: CCGACGTTAATCTGAAAGGAA, siCEBPA: GCTGGAGCTGACCAGTGACAA; siCEBPD: GCCGACCTCTTCAACAGCAAT; A non-targeting, scramble silencing RNA was used as control (shCtrl). Virus packaging was performed in 293T cells after co-transfection of packaging plasmids (pRRE, pCMV-VSVG, and pRSV-REV, Addgene) using Lipofectamine 3000.

### Enzyme-linked immunosorbent assay (ELISA)

CAFs were transfected with lentivirus CD36 and control NC vector, respectively. The same number of the transfected CAFs were cultured in α-MEM with 10% FBS until 80% of confluency. These transfected CAFs were washed with PBS and cultured in the serum-free media. Supernatants were harvested 48 h later and used for subsequent ELISA assays. The OxLDL, iNOS, IL-6 and MIF ELISA kits were purchased from Invitrogen (88-7066) and AbFrontier (LF-EK50529), and the experiments were performed according to the manufacturer’s instructions.

### ChIP

DNA and associated proteins on chromatin in cultured cells were crosslinked by 1% formaldehyde for 15 min at 37 °C. Cells were then scraped and collected in cellular lysis buffer (5 mM Pipes, 85 mM KCl, 0.5% NP-40, and protease inhibitors). Cytoplasmic lysates were discarded and nuclear components were resuspended in nuclear lysis buffer (50 mM Tris pH 8, 10 mM EDTA pH 8, 0.2% SDS, and protease inhibitors), and sonicated for 10 min (Covaris). Approximately 4 mg of CEBPA (Cat# sc-365318, Santa cruz), CEBPD (Cat# sc-365546, Santa Cruz Biotechnology) or control IgG (Cat# ab6715, Abcam) were incubated with 25 mL of protein G magnetic beads for 6 h at 4 °C, and then incubated with 100 mg of cleared chromatin overnight at 4 °C. After three washes, immunoprecipitated material was eluted at 55 °C for 1 h with 10 µg/mL proteinase K, and then decrosslinked at 65 °C for 4 h. The primer sequences used for ChIP-qPCR were listed in Supplementary Table S4.

### RNA extraction and real-time qPCR analysis

Total RNA was isolated using FastPure Blood/Cell/Tissue/Bacteria DNA Isolation Mini Kit (Vazyme Biotech Co., Ltd; Catalog No: DC112). First-strand cDNA was generated using the GenFQ III Reverse Transcriptase (Genfine Biotech Co., Ltd; Beijing, China; Catalog No: A107) according to the manufacturer’s protocol. Realtime-qPCR was performed in the StepOne Real-Time PCR System (Applied Biosystems) using GenFQ SYBR qPCR Master Mix (Genfine Biotech Co., Ltd; Beijing, China; Catalog No: A104) and the gene-specific primers shown in Supplementary Table S4. *GAPDH* was employed as an endogenous control for mRNAs. The relative expression of RNAs was calculated using the comparative Ct method. The primer sequences used were shown in Supplementary Table S4.

### Western blot

Cell lysates and supernatants were resolved by electrophoresis, transferred to a polyvinylidene fluoride membrane, and probed with antibodies against β-tubulin (Cat# 2128, Cell Signaling Technology), iNOS (Cat# ab178945, Abcam), CD36 (Cat# ab252922, Abcam), CEBPA (Cat# sc-365318, Santa cruz), CEBPD (Cat# sc-365546, Santa Cruz Biotechnology), MIF (Cat# ab187064, Abcam), p-p38 (Cat# ab195049, Abcam), p65 (Cat# ab32536, Abcam), SOX2 (Cat# ab92494, Abcam), OCT4 (Cat# ab181557, Abcam), STAT3 (Cat# ab68153, Abcam), or HLA-DRA (Cat# ab92511, Abcam). The antibodies were listed in the Supplementary Table S5.

### MIF staining assay

Multiplex staining of was performed using TSA 7-color kit (D110071-50T, Yuanxibio), according to manufacturer’s instruction. Primary antibodies included four panels: the first panel was CD36 (Cat# ab252922, Abcam), ACTA2 (Cat# ab7817, Abcam), CD33 (Cat# ab269456, Abcam), CD11b (Cat# ab133357, Abcam); the second panel was HLA-DRA, ACTA2, MCAM (Cat# ab75769, Abcam), CD36, FAP (Cat# ab207178, Abcam); the third panel was ACTA2, CD36, MYH11 (Cat# ab224804, Abcam), LUM (Cat# ab168348, Abcam) and MIF (Cat# ab187064, Abcam). Primary antibodies were sequentially applied, followed by horseradish peroxidase-conjugated secondary antibody incubation (1:1, Cat# DS9800, Lecia Biosystems; 1:1 Cat# A10011-6/A10012-6, WiSee Biotechnology), and tyramide signal amplification (M-D110051, WiSee Biotechnology). The slides were microwave heat-treated after each TSA operation. Nuclei were stained with DAPI (D1306, ThermoFisher) after all the antigens above were labeled. The stained slides were scanned to obtain multispectral images using the Pannoramic MIDI imaging system (3D HISTECH). Five randomly selected tumor regions from each patient were counted for the number of target cells by HALO Software (Indica Labs). The antibodies were listed in the Supplementary Table S5.

### IHC assay

After deparaffinization, slides were hydrated in alcohol and endogenous peroxidase activity was quenched for 30 min in 10% hydrogen peroxide. Antigen epitope retrieval was induced by microwave heating. To examine the expression pattern of candidate antibodies in HCCs and adjacent tissues, sections were immunostained with primary antibodies overnight at 4 °C, the secondary antibody used for immunostaining was biotin-conjugated anti-rabbit or anti-mouse immunoglobulin (Beijing Zhongshan Biotechnology). The signal was detected using an ABC kit (Beijing Zhongshan Biotechnology), following the protocol of manufacturer. Hematoxlin was used for counterstaining. The antibodies were listed in the Supplementary Table S5.

### Animal studies

All research involving animals were complied with protocols approved by the Fudan University Animal Care and Use Committee. Six- to eight-week-old male C57BL/6J mice were treated under the following conditions. CAFs or CD36^kd^ CAFs were generated by specific CD36-shRNA or Control-shRNA. For orthotopic HCC model, 6 × 10^6^ MHCC97H or Hep1-6 cells alone or with 2 × 10^5^ CAFs or CD36^kd^ CAFs were resuspended in 50 μL growth-factor-reduced matrigel (#354234, Corning) and injected into the liver lobe of anesthetized 6-week-old male nude mice. Anti-Gr-1 antibodies (Clone 1A8, 200 g/mouse, Bio-Xcell) were intraperitoneally (i.p.) injected every 5 days starting at one day before cell injection. For in vivo tumorigenicity assay, MHCC97H or Hep1-6 cells alone or together with CAFs at a ratio of 30:1 subcutaneously (s.c.) inoculated into nude or immunocompetent mice. SSO (15 mg/kg) was i.p. injected every day starting at one day after cell inoculation. To establish HCC spontaneous model, we injected 2 mL PBS containing *CTNNB1-N90/sgTP53* plasmid and sleeping beauty transposon (10 μg per mice) into mice by HDTVi. For the treatment regime, anti-PD-1 (5 mg/kg) was i.p. injected every two days or SSO (15 mg/kg) every two days starting from day 7 after tumor cell implantation or plasmid injection. While for anti-PD-1 resistant murine HCC model, we firstly chose anti-PD-1 mice group, which administered anti-PD-1 (i.p. 15 mg/kg, every two days) for continue 50 days when firstly administered anti-PD-1 (i.p. 5 mg/kg, every two days) for 24 days. Then, SSO (15 mg/kg) was injected every two days starting from day 61. We then sacrificed mice when administered anti-PD-1 at day 31 and day 80, respectively. Tumor burden, and proportion of Tregs and MDSCs were evaluated.

### Tumor sphere formation assay

Tumor cells were suspended in the sphere formation medium supplemented with serum-free DMEM-F12 (GIBCO) containing B27 (1:50, #17504044, Invitrogen), human recombinant EGF (20 ng/mL, #AF-100-15, PeproTech), and insulin (4 mg/mL, #11376497001, Roche) and then added to ultra-low adsorption cell culture plate with 500 cells per well. Different types of CM from MDSCs and CD36^+^ CAFs or CD36^kd^ CAFs were added into sphere formation medium at the volume ratio of 1:1 without or with each of the following inhibitors: BIRB 796 (5 M, #HY-10320), ISO-1 (10 nM, # HY-16692) and Stattic (100 nM, HY-13818). Tumor spheres with diameter > 75 mm were calculated under microscope after 14-day culture. Tumor sphere formation efficiency was calculated by dividing the number of spheres by the original number of single tumor cell seeded.

### Preparation of conditioned media (CM) and cell stimulation

CD36^+^ CAFs or CD36^kd^ CAFs (5 × 10^5^ cells/well at 12-well plate), CD33^+^ MDSCs (2 × 10^5^ cells/well at 48-well plate) and HCC cells (5 × 10^5^/well at 12-well plate) were cultured in serum-free DMEM-F12 or RPMI-1640 with 1% FBS for 24 h. To obtain CM of CAFs-educated CD33^+^ MDSCs, CM from CAF were used to culture blood CD33^+^ MDSCs for 6 h followed by culturing with fresh serum-free medium for another 24 h. All CM were filtered and used for further study. Blood CD33^+^ MDSCs were stimulated with MIF (20 ng/mL, #HY-P7387, MedChemExpress), CM from tumor cells, CD36^+^ CAFs or CD36^kd^ CAFs (the ratio of volume: 1:1) without or with neutralizing ISO-1 (10 nM, #HY-P7387, MedChemExpress) and/or Stattic (100 nM, HY-13818, MedChemExpress) for 24 h, respectively.

### Uptake of fatty acids or lipoproteins, lipid peroxidation assay

For measuring uptake of fatty acids or cholesterol, cells were incubated in PBS containing 0.5 mg/mL C1-BODIPY 500/510 C12 (ThermoFisher, D3823), or PBS containing 0.1 mg/mL BODIPY FL C16 (ThermoFisher, D3821) for 20 min at 37 °C. For measuring uptake of cholesterol, cells were incubated in PBS containing NBD Cholesterol (ThermoFisher, N1148) at a final concentration of 10 mM for 15 min at 37 °C. For measuring LDL uptake, cells were incubated in PBS containing 0.3% BSA and 20 mg/mL BODIPY FL LDL (ThermoFisher, L3483) for 30 min at 37 °C. For measuring OxLDL uptake, cells were incubated in PBS containing OxLDL-DyLightTM-488 (1:20 dilution, Oxidized LDL Uptake Assay Kit, Cayman Chemical, #601180), or PBS containing 50 mg/mL DiI-labeled human high oxidized low density lipoprotein (Kalenbiomed, Cat# 770262-9) for 30 min at 37 °C. After incubation, cells were washed with MACS buffer (PBS containing 2% FBS) for surface staining. For measuring lipid peroxidation, cells were incubated in PBS containing 2 mM BODIPY 581/591 C11 reagent (ThermoFisher, C10445) for 30 min at 37 °C before live dead and surface staining. CD36^+^ CAFs or CD36^kd^ CAFs were sorted prior to C11 lipid peroxidation assay to avoid interference of tumor cells in the assay. The reagents were listed in the Supplementary Table S5.

### Nuclear/cytoplasmic protein fractionation

Nuclear and cytoplasmic fractionation was performed according to the Kit protocols from Abcam (ab113474). After centrifuged, the nuclear and the cytosolic fraction were collected respectively. Equal volume of the nuclear and cytoplasmic lysates were tested by western blot.

### Flow cytometry

Briefly, four different group tumor tissues were digested at 37 °C for 30 min with 1 mg/mL Collagenase D and 0.1 mg/mL DNase I (Roche). Digestion was stopped by EDTA and cells were filtrated through 70 mm cell strainers and washed twice with PBS containing 1 mM EDTA and 2% FBS (staining buffer). Cells were re-suspended in the staining buffer and stained with following antibodies on ice for 30 min: anti-CD45, anti-CD8, anti-IFNg, anti-Granzyme B, anti-CD11b, anti-CD33, anti-FOXP3, anti-CD25, anti-F4/80, Ly6C were purchased from BioLegend. For intracellular staining, cells were fixed with fixation buffer (Biolegend) on ice for 15 min, and then washed twice with Intracellular Staining Permeabilization Wash Buffer (Biolegend). Antibodies against IFN-g (Clone XMG1.2) and Granzyme B (Clone: QA16A02) were added and incubated for 1 h on ice. The cytokine producing cells were determined by flow cytometry. The flow cytometry data were collected on Fortessa (BD) and analyzed by FlowJo (Tree Star). For cell sorting, CD8^+^ T cells that were co-cultured with tumor cells for 6 h were collected and washed with culture medium. Re-suspended cells were stained with anti-CD8a antibodies (Clone: 53-6.7) for 30 min on ice. After a washing step, cells were sorted on a BD FACS AriaIII (BD) and lysed in the buffer RLT plus (QIAGEN).

### Statistical analysis

All purchased mice in this study had similar age. Male and female mice were included in similar numbers for each animal experiment. Multiple-group comparisons were performed by one-way or two-way ANOVA followed by a Tukey correction to compare each group. All data are reported as mean ± SD. Statistical analysis was performed with a 2-tailed *t*-test using GraphPad Prism software and R language (https://www.R-project.org/). *P* < 0.05 was considered statistically significant. All in vitro experiments were performed with at least three biological replicates.

## Supplementary information

supplementary information

## Data Availability

The raw scRNA-seq data we used to extend our analyses on fibroblasts have been uploaded to the National Center for Biotechnology Information’s Gene Expression Omnibus database repository (https://www.ncbi.nlm.nih.gov/geo) under accession number GEO: GSE202642. The sample identifiers from the scRNA-seq dataset (GSE202642) are listed below: GSM6127499 (sample T1, related to Table S1), GSM6127500 (sample T3), GSM6127501 (sample T2), GSM6127502 (sample T5), GSM6127503 (sample T4), GSM6127504 (sample T7), and GSM6127505 (sample T6). The raw and processed mouse scRNA-seq data have been deposited in the GSA database with the accession numbers CRA026592 (raw) and OMIX010443 (processed). In addition, we consulted other publicly available scRNA-seq datasets, including GEO: GSE156625 and GEO: GSE154170 and GEO: GSE149614 from the National Center for Biotechnology Information’s Gene Expression Omnibus database repository. Additional information related to the data in this study is available from the lead contact upon request.
