# Supplementary material for: CD36+ cancer-associated fibroblasts provide immunosuppressive microenvironment for hepatocellular carcinoma via secretion of macrophage migration inhibitory factor
Source: Cell Discov. 2023 Mar 6;9:25. doi: 10.1038/s41421-023-00529-z (PMC9988869; doi:10.1038/s41421-023-00529-z)
Supplement: Supplementary file 1 — supplementary information [file 41421_2023_529_MOESM1_ESM.pdf]

Supplementary figures

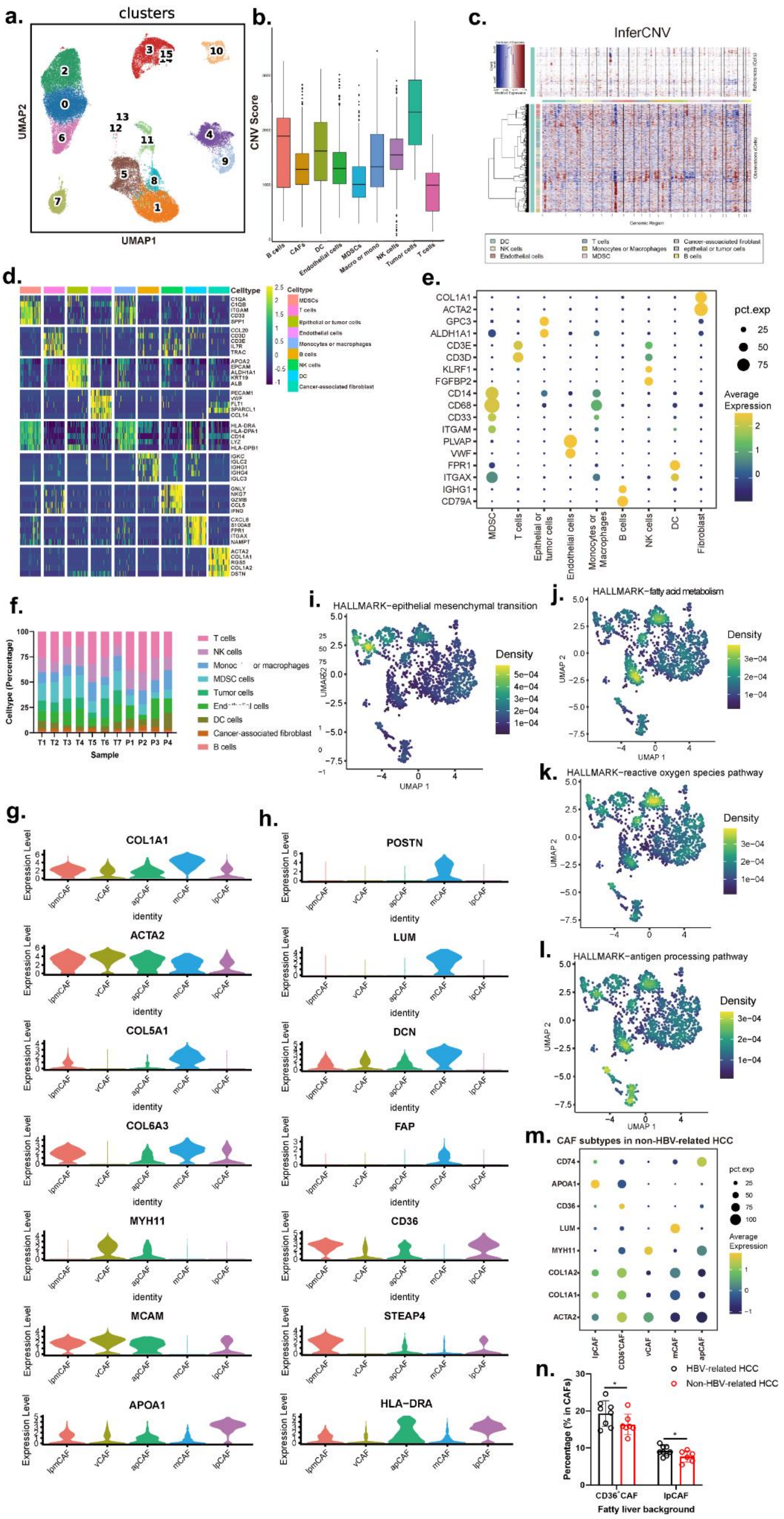

Supplementary Fig. S1. The marker genes and pathway activity of CAF subtypes in human HCC tumors.

(a) UMAP plots for the different clusters identification of single cells. (b) Box plots showing the CNV signals for each cell type. (c) Heatmap plots showing the CNV signals for each cell type. (d) Heatmap showing the top DEGs (Wilcoxon test) in each cell type. (e) Dotplots showing cell marker genes in each cell types. (f) Bar plots showing the proportion of cell types in each sample. (g-h) Violin plots showing gene markers in different CAF subtypes. (i-l) UMAP plots showing pathway activity for different human CAF subtypes. (m) Dotplots showing cell marker genes of each CAF subtypes in non-HBV-related HCC. (n) Box plot showing the proportions of CD36+CAFs and lpCAFs in fatty liver background of HBV-related HCC or non-HBV-related HCC by mIF. CNV, copy number variation; DEGs, differentially expressed genes; HCC, hepatocellular carcinoma; scRNA-seq, single-cell RNA-sequencing; UMAP, uniform manifold approximation and projection.

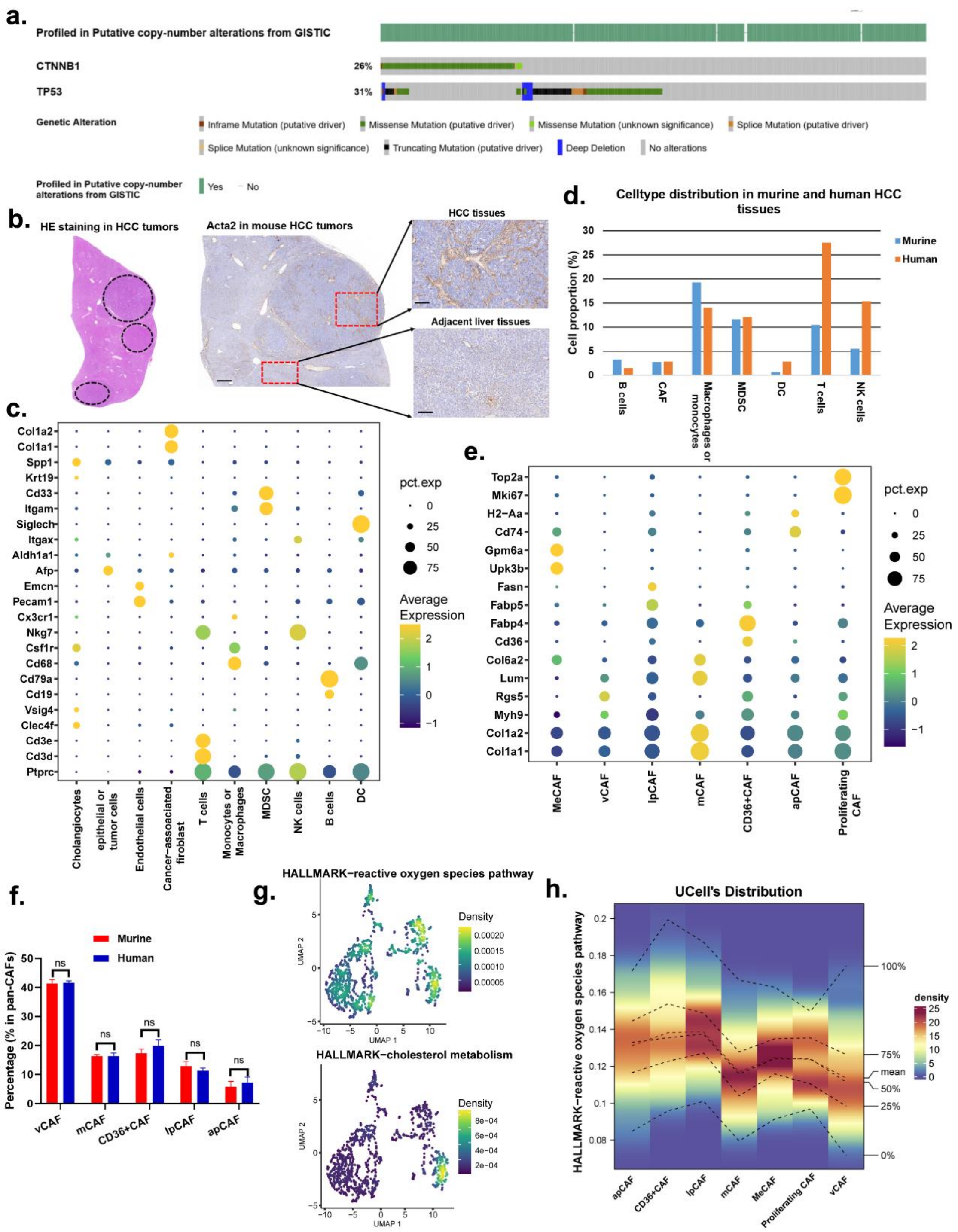

Supplementary Fig. S2. The marker genes and pathway activity of CAF subtypes in murine HCC tumors.

(a) The mutation landscape of CTNNB1 and TP53 in TCGA HCC databases. (b) HE staining and representative image of IHC for Acta2 in murine HCC tissues. (c) Dotplots show marker genes in different cell types in murine HCC tumors. (d) The

comparison of major cell types between human and murine HCC tumors. (e) Dotplots show marker genes in different CAF subtypes in murine HCC tumors. (f) The comparison of major CAF subtypes between human and murine HCC tumors. (g) UMAP plots show enriched pathways in human CAF clusters. (h) UCell's distribution in different CAF subtypes for murine HCC tumors.

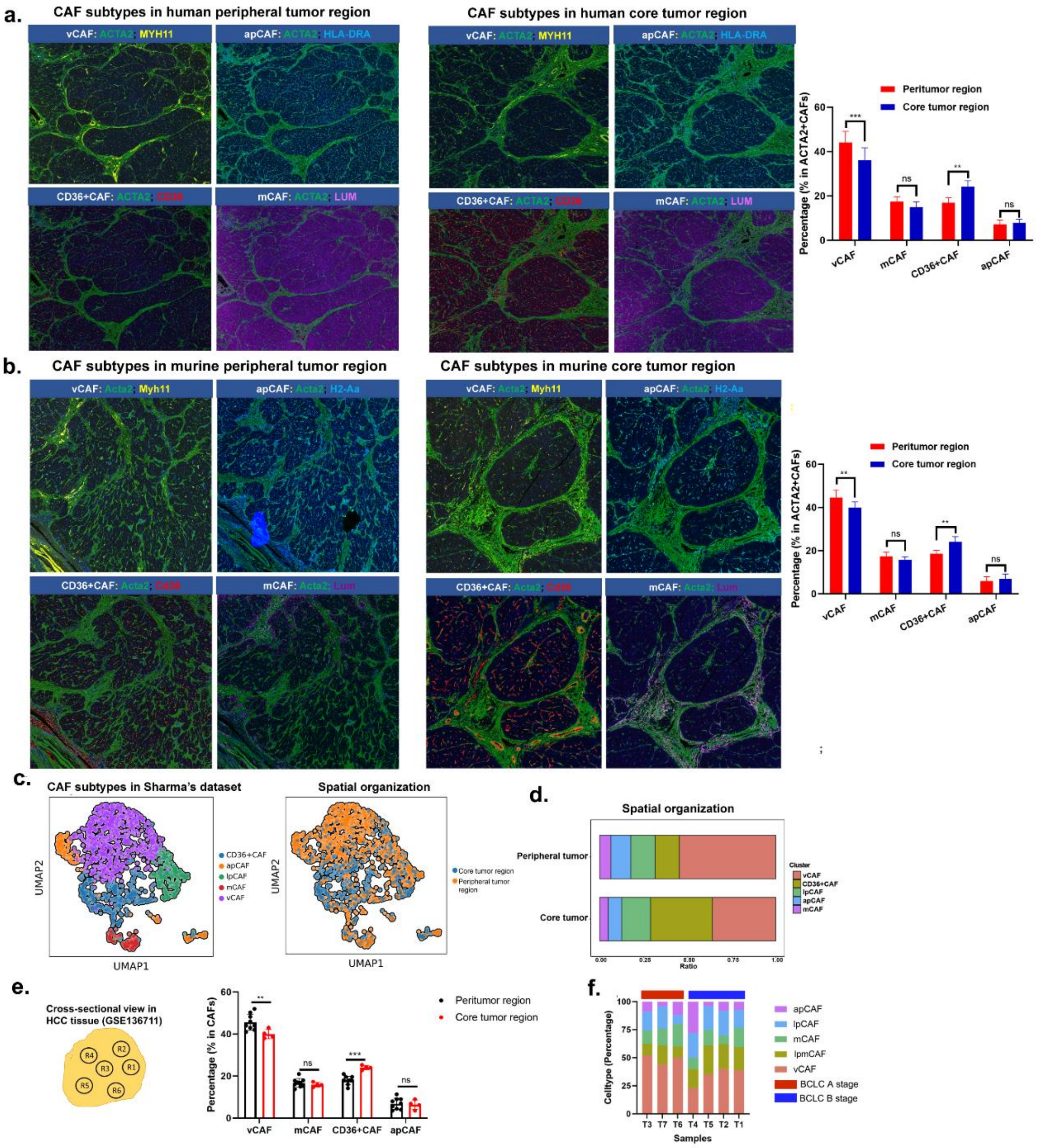

Supplementary Fig. S3. The CAF heterogeneity of spatial in human and mouse HCC tumors.

(a-b) Multiplex immunofluorescence staining shows spatial distribution of CAF subtypes in human (a) or murine (b) HCC tumors. (c-d) The spatial organization of CAF subtypes by Sharma's scRNA-seq dataset. (e) The spatial heterogeneity analysis of CAF subtypes in HCC tumors by CIBERSOTx. (f) Bar plots show CAF clusters in different tumor regions within HCC samples.

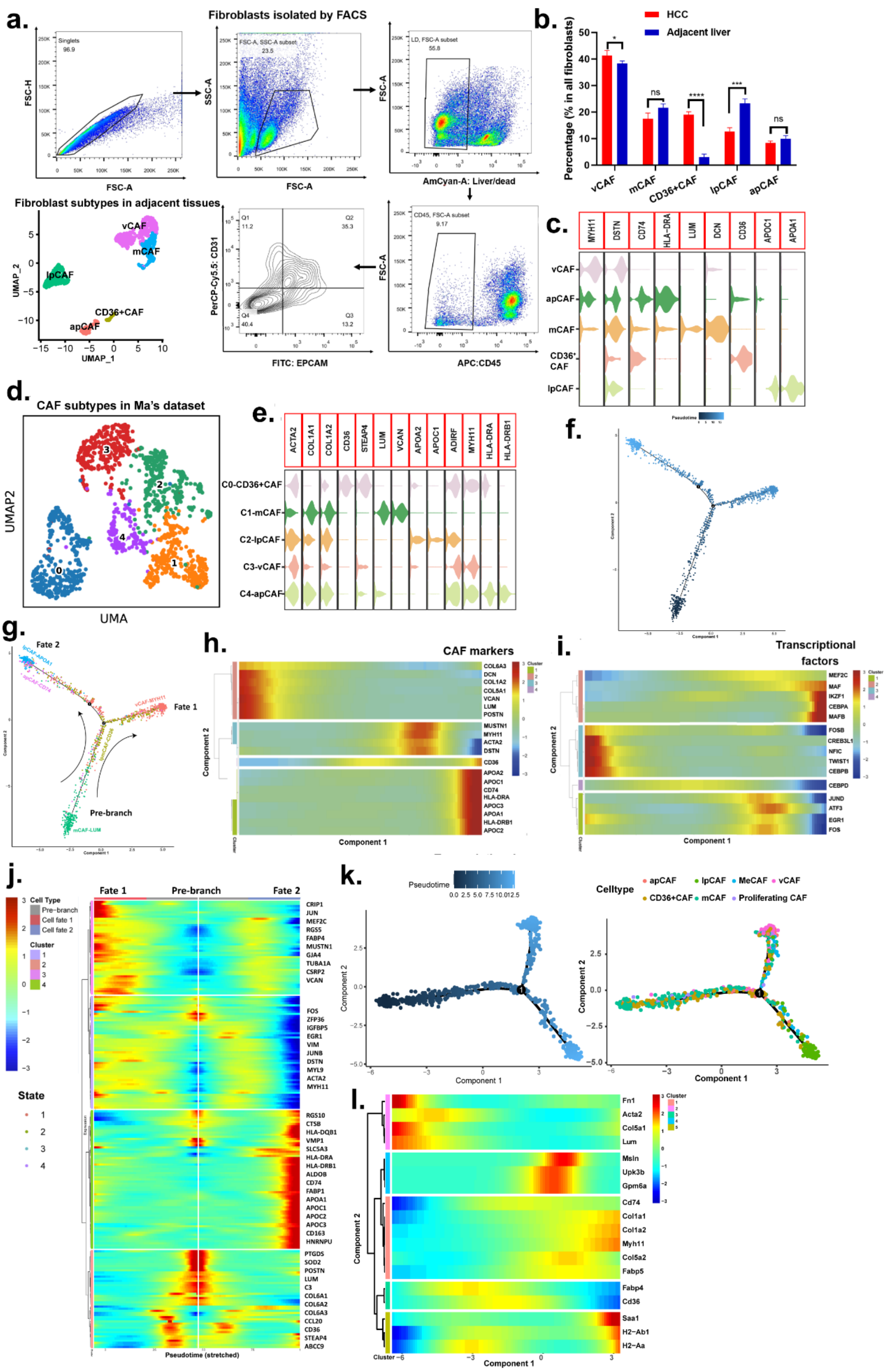

Supplementary Fig. S4. The CAF distribution and time trajectory of CAF subtypes in HCC tissues.

(a) The fibroblasts clusters in human adjacent liver tissues by FACS. (b) The comparison of fibroblasts clusters between HCC and adjacent liver tissues from scRNA-seq data by FACS. (c) Violin plot for CAF subtypes from adjacent liver tissues fron scRNA-seq by FACS. (d) The UMAP plot shows CAF subclusters in Ma et al.'s scRNA-seq data (GSE151530). (e) Violin plot for CAF subtypes in Ma et al.'s scRNA-seq data. (f-g) Trajectory of differentiation process colored by states and cell types. (h) Heatmap shows upregulated or downregulated CAF marker genes in the differentiation process. (i) Heatmap shows upregulated or downregulated transcriptional factors enriched in different CAFs in the differentiation process. (j) Heatmap shows upregulated or downregulated CAF marker genes in the differentiation process. (k) Trajectory of differentiation process colored by states and cell types for CAF subtypes from murine HCC tumors. (l) Heatmap shows upregulated or downregulated CAF marker genes in the differentiation process for murine HCC tumors.

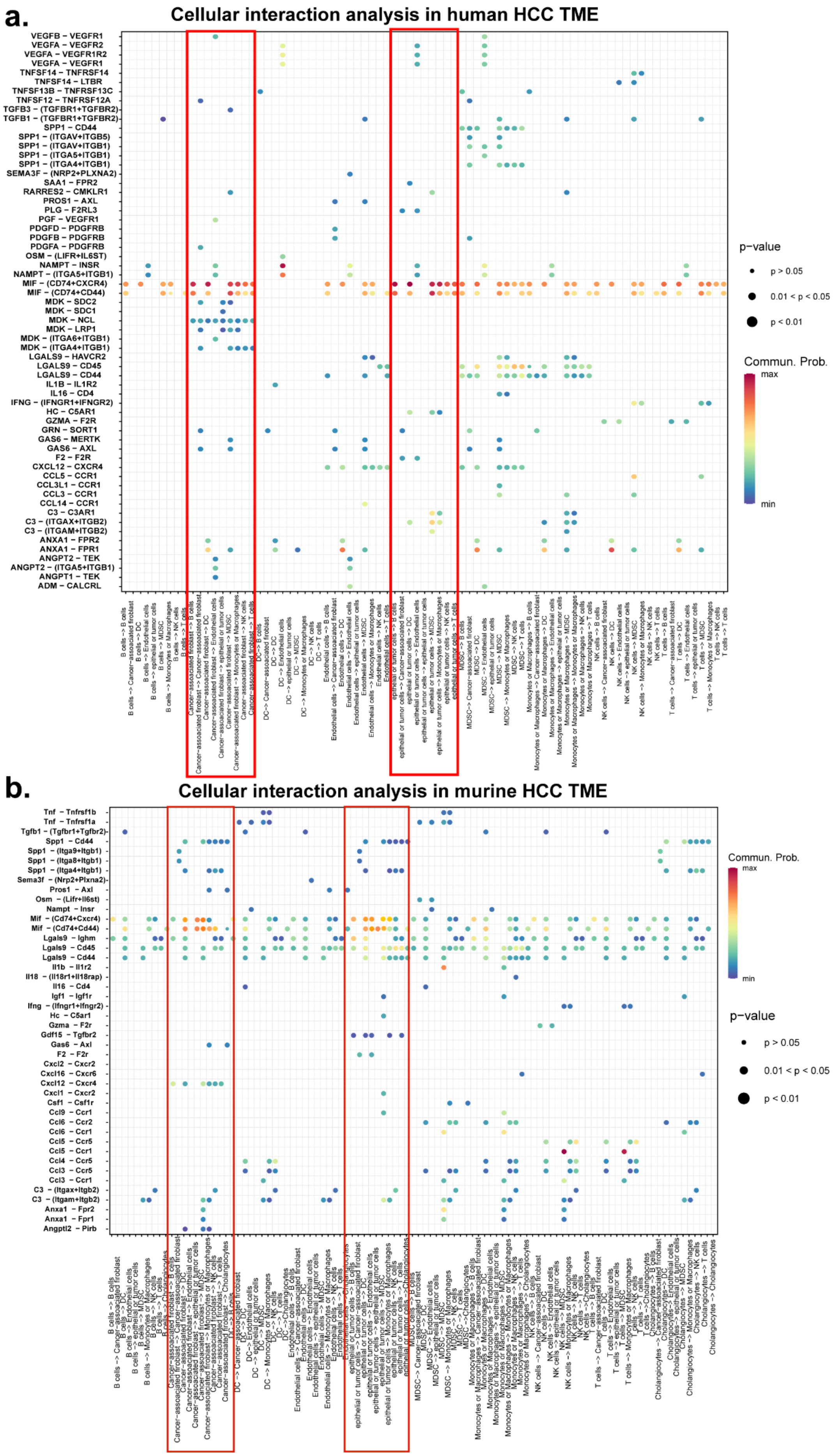
a.
Cellular interaction analysis in human HCC TME
p-value
p > 0.05
0.01 < p < 0.05
p < 0.01
Commun. Prob.
max
min
b.
Cellular interaction analysis in murine HCC TME
Commun. Prob.
max
min
p-value
p > 0.05
0.01 < p < 0.05
p < 0.01

Supplementary Fig. S5. Cell interaction analysis between all distinct cell types in human or murine HCC microenvironment.

(a) Dotplot shows pair-ligands between all distinct cell types in human HCC tumors. Red box indicates CAFs and tumor cells acting on other cell types.

(b) Dotplot shows pair-ligands between all distinct cell types in murine HCC tumors. Red box indicates CAFs and tumor cells acting on other cell types.

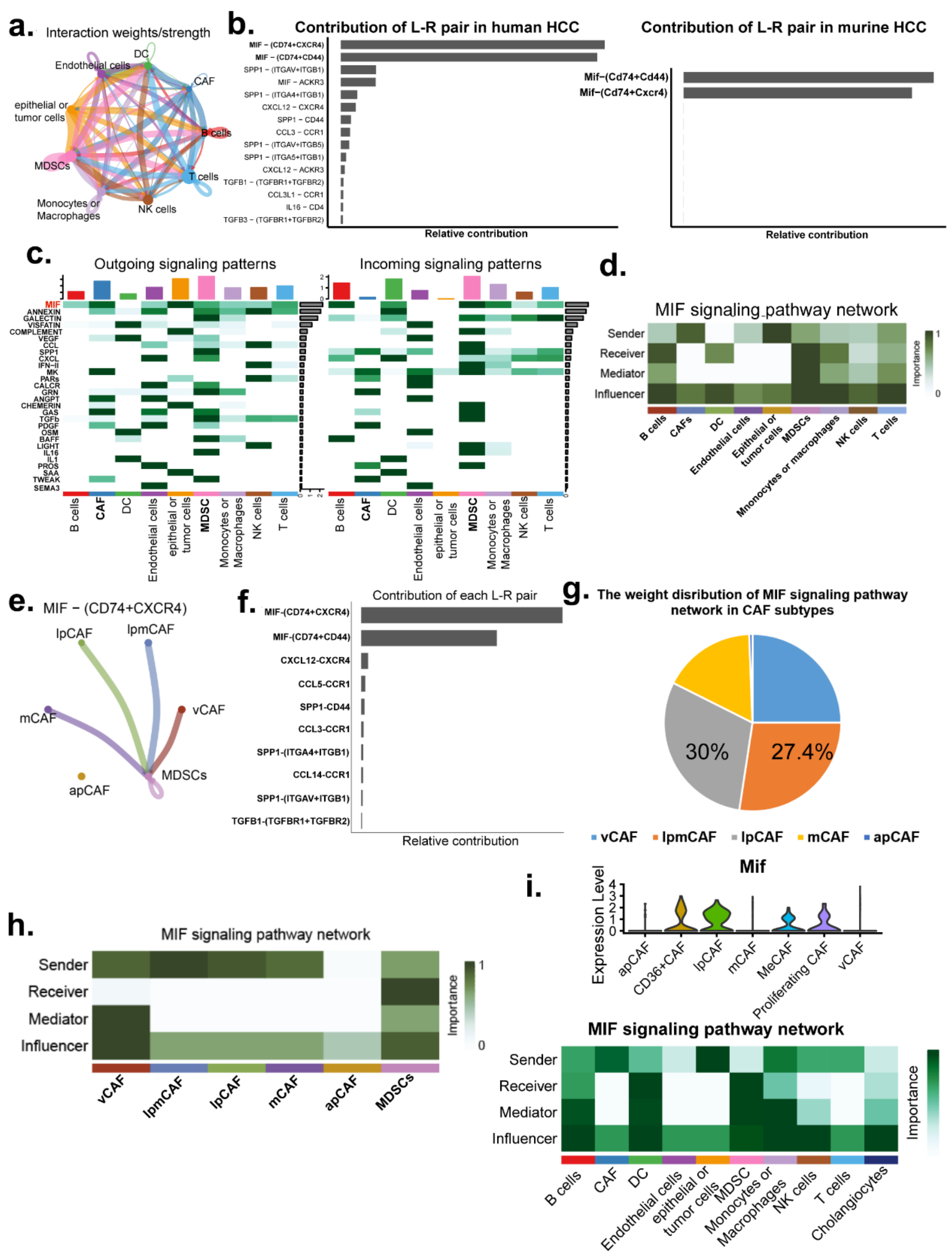

Supplementary Fig. S6. Cell interaction analysis between CAF and other distinct cell types.

(a) The interaction weights/strength between different cell types. (b) The contribution of ligand-receptor pair in human and murine HCC tumors. (c) Heatmap shows genes involving pair-ligands in different cell types for human HCC tumors. (d) MIF

signaling pathway network in all cell types for human HCC tumors. (e) MIF signaling pathway network between CAF subtypes and MDSCs in human HCC tumors. (f) The contribution of each L-R pair between CAF subtypes and MDSCs. (g) The weight distribution of MIF signaling pathway network between CAF subtypes and MDSCs. (h) MIF signaling pathway network between CAF subtypes and MDSCs in human HCC tumors. (i) MIF signaling pathway network and expression between all cell types in murine HCC tumors.

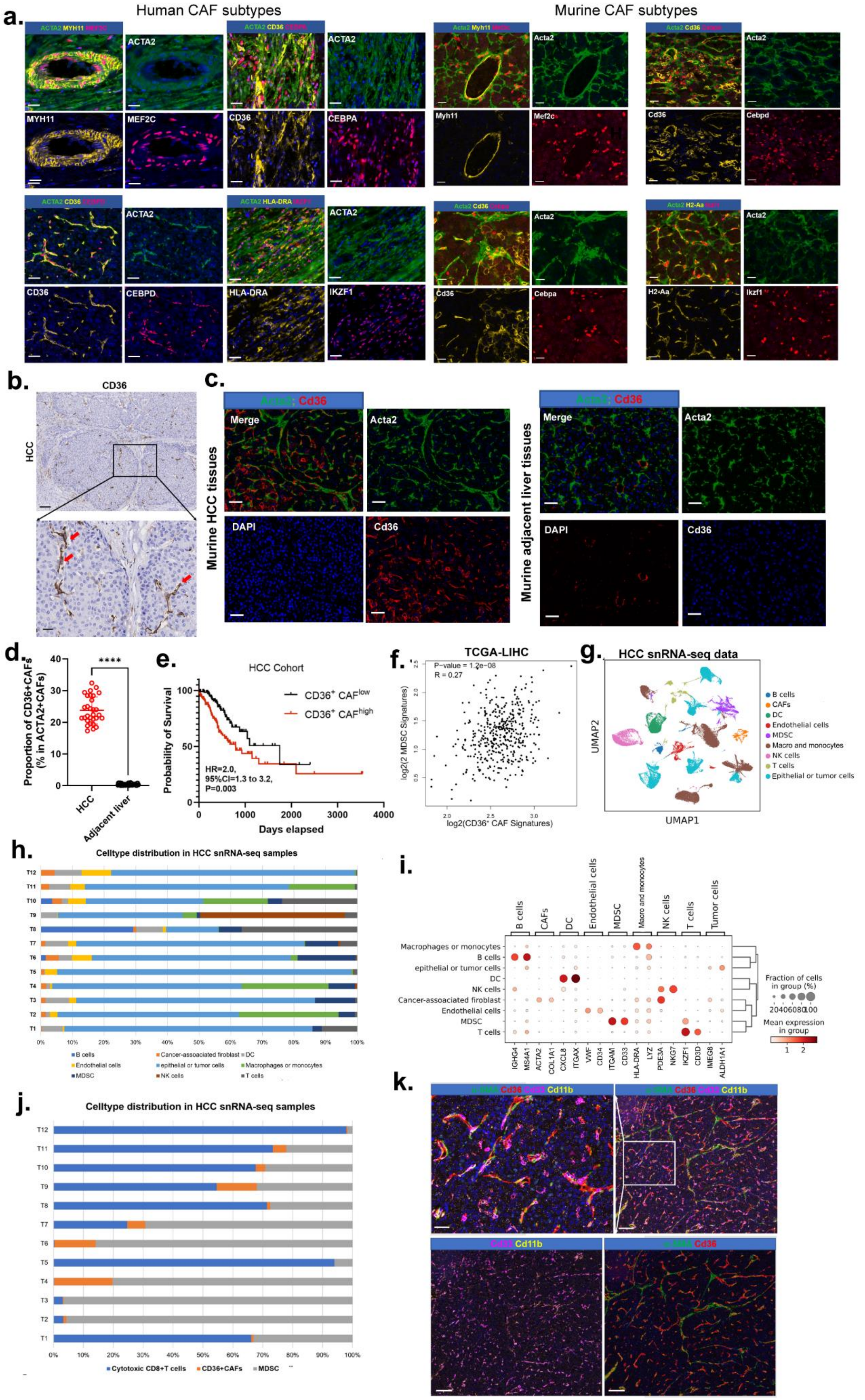
a.
Human CAF subtypes
Murine CAF subtypes
ACTA2
MYH11
MEF2C
CD36
CEBPA
CEBPD
HLA-DRA
IKZF1
Acta2
Myh11
Mef2c
Cd36
Cebpd
Cebpa
H2-Aa
Ikzf1
b.
CD36
HCC
c.
Murine HCC tissues
Murine adjacent liver tissues
Merge
DAPI
d.
Proportion of CD36+CAFs (% in ACTA2+CAFs)
HCC
Adjacent liver
e.
HCC Cohort
Probability of Survival
Days elapsed
HR=2.0, 95%CI=1.3 to 3.2, P=0.003
f.
TCGA-LIHC
P-value = 1.2e-08
R = 0.27
log2(2 MDSC Signatures)
log2(CD36+ CAF Signatures)
g.
HCC snRNA-seq data
UMAP1
UMAP2
B cells
CAFs
DC
Endothelial cells
MDSC
Macro and monocytes
NK cells
T cells
Epithelial or tumor cells
h.
Celltype distribution in HCC snRNA-seq samples
Cancer-assoaciated fiboblast
epithelial or tumor cells
Macrophages or monocytes
i.
Macrophages or monocytes
epithelial or tumor cells
Cancer-assoaciated firoblast
Tumor cells
Fraction of cells in group (%)
Mean expression in group
IGHG4
MS4A1
COL1A1
CXCL8
ITGAX
VWF
CD34
ITGAM
CD33
LYZ
PDE3A
NKG7
CD3D
IMEG8
ALDH1A1
j.
Celltype distribution in HCC snRNA-seq samples
Cytotoxic CD8+T cells
CD36+CAFs
k.
Cd36
Cd11b

Supplementary Fig. S7. CAF subtype-specific transcription factors and the interactions between different CAFs and MDSCs.

(a) Multiplex immunofluorescence staining showed top expressed transcriptional factors in different CAF subtypes from murine and human HCC tumors. (b) CD36 expression in HCC tissues by IHC experiments. (c) Multiplex immunofluorescence staining shows $CD36^+$ CAF was infiltrated less in adjacent tissues. (d) $CD36^+$ CAFs infiltrated more in HCC tissues than adjacent tissues. ∗P <0.05, ∗∗P <0.01, ∗∗∗P <0.001 by Student's t-test. (e) High proportion of $CD36^+$ CAFs predict worse prognosis in HCC patients. (f) $CD36^+$ CAFs correlate with MDSCs in TCGA datasets. (g) UMAP plot showed distinct cell types in HCC snRNA-seq data. (h) The dotplot showed specific gene markers in distinct cell types from HCC snRNA-seq data. (i) Celltype distribution in HCC snRNA-seq samples. (j) The cytotoxic CD8+ T cells, CD36+CAFs and MDSCs in different snRNA-seq samples. (k) Multiplex immunofluorescence staining shows $CD36^+$ CAF interacted with MDSCs in murine HCC tissues.

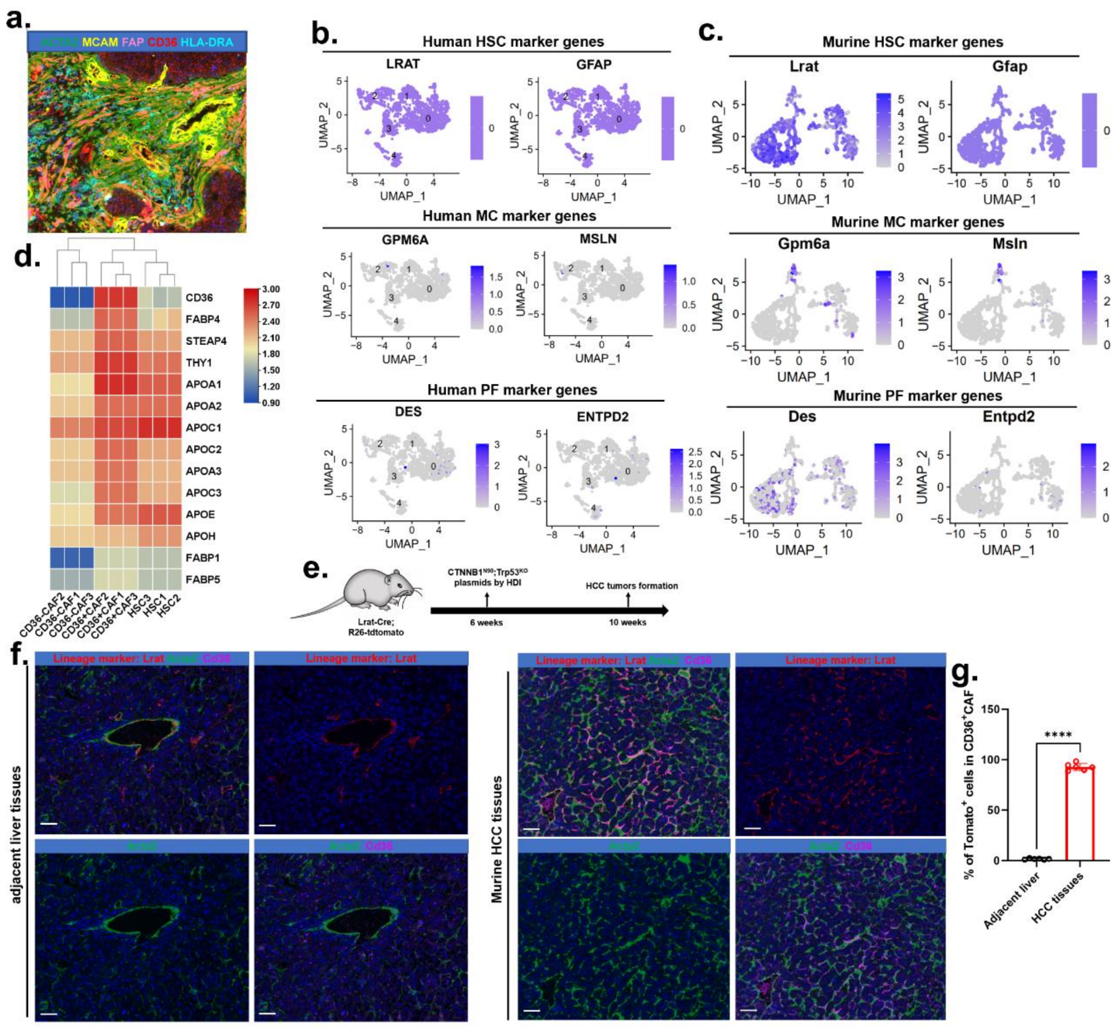

Supplementary Fig. S8. The cellular origin of CD36+CAFs in murine HCC models. (a) The cell surface markers of CAF subtypes in HCC tumors. (b) The expression of specific genes for hepatic stellate cells, mesenchymal cell, portal fibroblasts in human CAF scRNA-seq data. (c) The expression of specific genes for hepatic stellate cells, mesenchymal cell, portal fibroblasts in murine CAF scRNA-seq data. (d) Heatmap showed genes related to lipid metabolism highly expression in CD36+CAFs and hepatic stellate cells when compared with CD36-CAFs. (e) The schematic diagram showed established spontaneous HCC in Lrat-Cre; R26-tdtomato mice. (f-g) Multiplex immunofluorescence staining showed CD36+CAFs originated from hepatic stellate cells in HCC tumors. Data are mean ± s.d. of n=6 independent experiments. ****P <0.0001 by Student’s t-test.

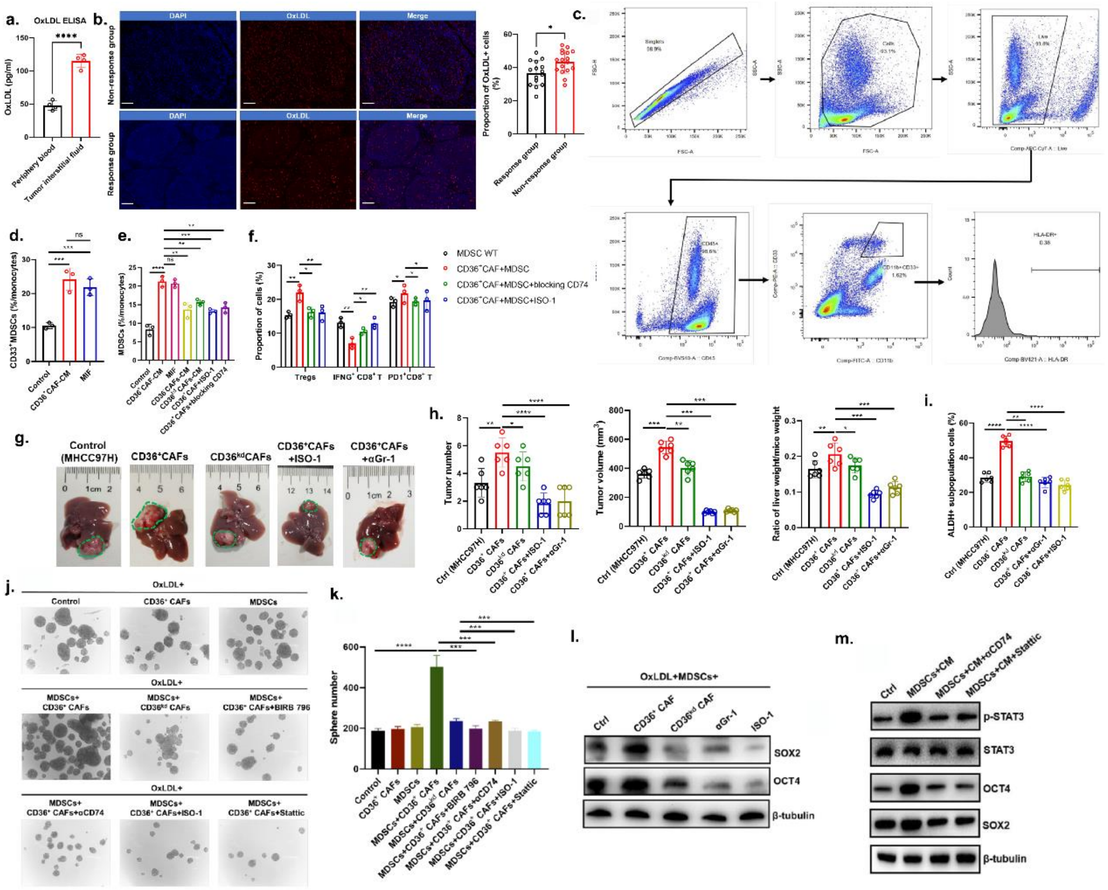

Supplementary Fig. S9. CD36+ CAF-derived MIF potentiates the capacity of MDSCs to promote tumor stemness in HCC.

(a) The levels of OxLDL between periphery blood and tumor interstitial fluid by ELISA assays. (b) The OxLDL+ cells between anti-PD-1 response and non-response group by IF assays. (c)The strategies for MDSCs by flow cytometery in human HCC tissues. (d) Human MDSC proportion was measured by flow cytometry when co-cultured with Ctrl, CD36+ CAFs, MIF recombinant protein. (e) Murine MDSC proportion was measured by flow cytometry when co-cultured with Ctrl, CD36+ CAFs, MIF, CD36- CAFs, CD36kd CAFs, a combination of CD36+ CAFs and MIF inhibitor ISO-1, or a combination of CD36+ CAFs and CD74 blocking agents. (f) The proportion of IFNG+ or PD1+CD8+ T cells and Tregs were evaluated in MDSCs-WT, CD36+CAFs+MDSCs, a combination of CD36+CAFs+MDSCs and ISO-1 or blocking CD74 by flow cytometry. (g) Representative images of HCC tumors from

the orthotopic HCC model. (h) Tumor numbers, tumor volume and ratio of liver weight and mice weight from the orthotopic HCC model. CAFs transduced with the empty lentiviral vector as a control. (i) ALDH+ cells of HCC nodules. CAFs transduced with the empty lentiviral vector as a control. (j-k) Representative images and tumorsphere formation efficiencies. (l) Western blotting assay show stemness marker genes for different treating groups. (m) Western blotting assay show stemness marker genes and STAT3 expression for different treating groups. Data shown as mean ± S.E.M., one-way ANOVA following multiple comparison test was used, ***p < 0.001, ** p < 0.01, *p< 0.05, and ns = not significant.

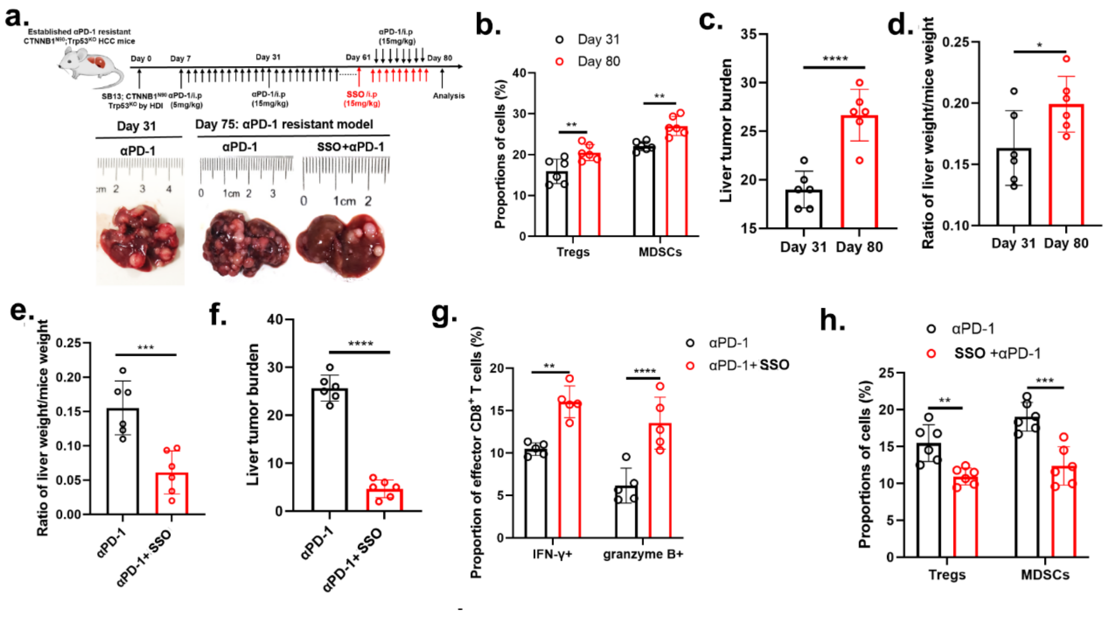

Supplementary Fig. S10. Targeting CD36 synergizes with immunotherapy in different HCC murine models.

(a) CD36 inhibitor SSO sythesizes PD-1 blockade in C57/BJ6 anti-PD-1 resistant spontaneous HCC model. (b) The changes of Tregs and MDSC in Day 31 and Day 81 HCC spontaneous murine model. (c-d) The tumor burden and ratio of tumor weight and mice weight in Day 31 and Day 81 HCC spontaneous murine model. (e-f) The

tumor burden and ratio of tumor weight and mice weight in anti-PD-1 and anti-PD-1 combined with SSO from HCC spontaneous murine model. (g-h) The changes of Tregs, MDSCs, IFN-γ^+, GZMB$^+$ CD8$^+$ T cells in two groups. Data shown as mean ± S.E.M., one-way ANOVA following multiple comparison test was used, ***p < 0.001, ** p < 0.01, *p< 0.05, and ns = not significant.

Supplementary tables

Supplementary Table S1. The baseline characteristics of 7 HBV-related HCC patients.

**Sample ID**	T1	T2	T3	T4	T5	T6	T7
**Age**	66	54	58	64	77	41	54
**Gender**	Male	Male	Male	Male	Male	Male	Male
**HBsAg**	Positive	Positive	Positive	Positive	Positive	Positive	Positive
**Diagnosis**	HCC	HCC	HCC	HCC	HCC	HCC	HCC
**Tumor number**	2	2	1	2	2	1	1
**Tumor diameter (cm)**	17*16/14*12	10*8/3.7*3	3.9*3.2	8*4.5/3.8*2.5	7*6/4.5*3	3.5*3.5	3.5*2.9
**Tumor grade**	III	II	III	II	II	II	II
**Tumor encapsulation**	Yes	No	Yes	Yes	No	No	Yes
**Microvascular invasion**	Yes	Yes	No	No	Yes	Yes	Yes
**Macrovascular invasion**	No	No	No	No	No	No	No
**GPC3 status**	Positive	Positive	Positive	Negative	Positive	Negative	Positive
**Liver**	Yes	Yes	Yes	No	Yes	No	Yes

**cirrhosis**							
**BCLC stage**	B	B	A	B	B	A	A

Supplementary Table S2. The list of cell markers in human HCC scRNA-seq data.

**Cell types**					**Top10 cell markers**				
**MDSC**	SPP1	RNASE1	C1QA	C1QB	LGMN	SLC40A1	C1QC	CTSB	CD33	ITGAM
**T cells**	CCL20	LTB	IL7R	CD2	TRAC	TRBC2	IL32	CD3D	KLRB1	BCL11B
**Epithelial or tumor cells**	APOA2	RBP4	ORM1	EPCAM	AMBP	APOC2	ALB	H19	CLU	AHSG
**Endothelial cells**	STC1	PLVAP	SPRY1	SPARCL1	PECAM1	TM4SF1	CALCRL	FLT1	VWF	CCL14
**Mono or Macro**	HLA-DRA	HLA-DPA1	CST3	HLA-DPB1	LYZ	CD74	AIF1	HLA-DQA1	C1QB	HLA-DRB1
**B cells**	IGKC	IGLC2	IGHG1	IGLC3	IGLC1	IGHG2	IGHA1	IGHG3	JCHAIN	IGHG4
**NK cells**	GNLY	NKG7	GZMB	CCL5	IFNG	KLRD1	FGFBP2	GZMH	CCL4	CST7
**DC**	CXCL8	S100A8	G0S2	ITGAX	NAMP	LUCAT	BASP1	FPR1	FCGR3	PTGS2

					T	1			B	
**CAFs**	ACTA2	TAGLN	RGS5	COL1A1	CALD1	COL1A2	COL3A1	MGP	IGFBP7	MYL9

Supplementary Table S3. The list of cell markers in murine HCC scRNA-seq data.

**Cell types**					**Top10 cell markers**					
**Cholangiocytes**	Clec4f	Sdc3	Cd5l	Psap	Dmpk	Lgmn	Igha	Slc40a1	Spp1	Clu
**Epithelial or tumor cells**	Serpina1e	Afp	Apoa1	Ahsg	Alb	Gstp1	Serpina1d	Serpina3k	Rbp4	Ttr
**Endothelial cells**	Pecam1	Emcn	Kdr	Igfbp7	Aqp1	Gpihbp1	Sparc	Ly6c1	Egfl7	Timp3
**CAFs**	Dcn	Col1a1	Col1a2	Col3a1	Sparc	Bgn	Col14a1	Igfbp7	Cxcl12	Igha
**T cells**	Ccl5	Trbc2	Cd3d	Cd3g	Nkg7	Hspa1b	Trac	Ms4a4b	Cd3e	Dnajb1
**Mono or Macro**	C1qb	C1qa	C1qc	Ctss	Lgals3	Ms4a6c	Lyz2	Vcam1	H2-Ab1	Cd68
**MDSC**	Cd33	Itgam	Retnlg	Ngp	Camp	Ltf	Cxcl2	G0s2	Il1b	Pglyrp1
**NK**	Gzma	Xcl1	Gzm	Il2rb	Nkg7	Ccl5	Klrk1	Klrb1c	Klre1	Ccl4

			b							
**B cells**	Igkc	Cd79a	Ighm	Ebf1	Iglc2	Cd74	Cd79b	H2-Aa	Iglc3	Ighd
**DC**	Siglech	Irf8	Klk1	Ccr9	Cox6a2	Rnase6	Gm21762	Tcf4	St8sia4	Iglc3

Supplementary Table S4. The list of PCR primers used in this study.

**ChIP assays primers sequences**	**Sequence (5'→3')**
CEBPD-MIF sense	CTGGCCAGGTAAGGCAACC
CEBPD-MIF anti-sense	TCCCTGTGCCCTATGAAAGC
CEBPA-MIF sense	AGCTGCAGGAACCAATACCC
CEBPA-MIF anti-sense	CAGGTGCCAGGCATACAAGA
IKZF1-CCL5 sense	TGGGAGAGACCCTATGACCA
IKZF1-CCL5 anti-sense	AACCAAGCATTGGCCGGTA
IKZF1-HLA-DRA sense	CAGGAGAGACCATGCTGTAGG
IKZF1-HLA-DRA anti-sense	AAGAGTGACCTGGCAGTGAGG
**Real-time qPCR primers sequences**	**Sequence (5'→3')**
**Human**	
β-actin sense	GGGAAATCGTGCGTGACATTAAG
β-actin anti-sense	TGTGTTGGCGTACAGGTCTTTG
MIF sense	TCTGCCATCATGCCGATGTT
MIF anti-sense	GCTCTTAGGCGAAGGTGGAG

ACTA2 sense	AAAAGACAGCTACGTGGGTGA
ACTA2 anti-sense	GCCATGTTCTATCGGGTACTTC
COL1A1 sense	GAGGGCCAAGACGAAGACATC
COL1A1 anti-sense	CAGATCACGTCATCGCACAAC
COL1A2 sense	GTTGCTGCTTGCAGTAACCTT
COL1A2 anti-sense	AGGGCCAAGTCCAACTCCTT
COL5A2 sense	GACTGTGCCGACCCTGTAAC
COL5A2 anti-sense	CCTGGACGACCACGTATGC
COL6A3 sense	ATGAGGAAACATCGGCACTTG
COL6A3 anti-sense	GGGCATGAGTTGTAGGAAAGC
COL5A1 sense	GCCCGGATGTCGCTTACAG
COL5A1 anti-sense	AAATGCAGACGCAGGGTACAG
PDGFRB sense	AGCACCTTCGTTCTGACCTG
PDGFRB anti-sense	TATTCTCCCGTGTCTAGCCCA
MYH11 sense	CGCCAAGAGACTCGTCTGG
MYH11 anti-sense	TCTTTCCCAACCGTGACCTTC
DSTN sense	ATTTTGTGGGAATGCTTCCTGA
DSTN anti-sense	GCATCCTTGGAGCTTGCATAG
MUSTN1 sense	CCCGAGGAAACCTGACCAAG
MUSTN1 anti-sense	CACTCTCGCATGACCTGGTAG
MCAM sense	AGCTCCGCGTCTACAAAGC
MCAM anti-sense	CTACACAGGTAGCGACCTCC

CD36 sense	GGCTGTGACCGGAACTGTG
CD36 anti-sense	AGGTCTCCAACTGGCATTAGAA
FABP4 sense	ACTGGGCCAGGAATTTGACG
FABP4 anti-sense	CTCGTGGAAGTGACGCCTT
STEAP4 sense	GGCTTTGGGAATACTTGGGTT
STEAP4 anti-sense	TGGACAAATCGGAACTCTCTCC
THY1 sense	ATCGCTCTCCTGCTAACAGTC
THY1 anti-sense	CTCGTACTGGATGGGTGAACT
APOA1 sense	CCCTGGGATCGAGTGAAGGA
APOA1 anti-sense	CTGGGACACATAGTCTCTGCC
APOA2 sense	CTGTGCTACTCCTCACCATCT
APOA2 anti-sense	CTCTCCACACATGGCTCCTTT
APOC1 sense	TCCAGTGCCTTGGATAAGCTG
APOC1 anti-sense	GGCTGATGAGTTCCCGAGC
APOC2 sense	TGTCCTCCTGGTATTGGGATTT
APOC2 anti-sense	TGTCTTCTCGTACAGGTTCTGG
LUM sense	TAACTGCCCTGAAAGCTACCC
LUM anti-sense	GGAGGCACCATTGGTACACTT
FAP sense	ATGAGCTTCCTCGTCCAATTCA
FAP anti-sense	AGACCACCAGAGAGCATATTTG
DCN sense	ATGAAGGCCACTATCATCCTCC
DCN anti-sense	GTCGCGGTCATCAGGAACTT

VCAN sense	GTAACCCATGCGCTACATAAAGT
VCAN anti-sense	GGCAAAGTAGGCATCGTTGAAA
POSTN sense	CTCATAGTCGTATCAGGGGTCG
POSTN anti-sense	ACACAGTCGTTTTCTGTCCAC
TIMP1 sense	CTTCTGCAATTCCGACCTCGT
TIMP1 anti-sense	ACGCTGGTATAAGGTGGTCTG
HLA-DRA sense	AGTCCCTGTGCTAGGATTTTTCA
HLA-DRA anti-sense	ACATAAACTCGCCTGATTGGTC
HLA-DRB1 sense	CGGGGTTGGTGAGAGCTTC
HLA-DRB1 anti-sense	AACCACCTGACTTCAATGCTG
CD74 sense	GACGAGAACGGCAACTATCTG
CD74 anti-sense	GTTGGGGAAGACACACCAGC
CCL5 sense	CCAGCAGTCGTCTTTGTCAC
CCL5 anti-sense	CTCTGGGTTGGCACACACTT
**Mouse**	
Mif sense	GAGGGGTTTCTGTCGGAGC
Mif anti-sense	GTTCGTGCCGCTAAAAGTCA
Acta2 sense	CCCAGACATCAGGGAGTAATGG
Acta2 anti-sense	TCTATCGGATACTTCAGCGTCA
Col1a1 sense	GCTCCTCTTAGGGGCCACT
Col1a1 anti-sense	ATTGGGGACCCTTAGGCCAT
Col1a2 sense	TCGTGCCTAGCAACATGCC

Col1a2 anti-sense	TTTGTCAGAATACTGAGCAGCAA
Col5a3 sense	CCCAAGCAAGTCAACATTATGGA
Col5a3 anti-sense	CGGTTCACAGTCAACCACCA
Col6a3 sense	GCTGCGGAATCACTTTGTGC
Col6a3 anti-sense	CACCTTGACACCTTTCTGGGT
Myh9 sense	AGAAGTTGGTATGGGTGCCTT
Myh9 anti-sense	CCCTGAGTAGTATCGCTCCTTG
Myh11 sense	ATGAGGTGGTCGTGGAGTTG
Myh11 anti-sense	GCCTGAGAAGTATCGCTCCC
Rgs5 sense	CGCACTCATGCCTGGAAAG
Rgs5 anti-sense	TGAAGCTGGCAAATCCATAGC
Mustn1 sense	GTCTAAGACATACCAGGTCATGC
Mustn1 anti-sense	GCGGCTGAATACAGATGGGG
Mcam sense	CCCAAACTGGTGTGCGTCTT
Mcam anti-sense	GGAAAATCAGTATCTGCCTCTCC
Cd36 sense	ATGGGCTGTGATCGGAACTG
Cd36 anti-sense	GTCTTCCCAATAAGCATGTCTCC
Fabp4 sense	AAGGTGAAGAGCATCATAACCCT
Fabp4 anti-sense	TCACGCCTTTCATAACACATTCC
Fabp5 sense	TGAAAGAGCTAGGAGTAGGACTG
Fabp5 anti-sense	CTCTCGGTTTTGACCGTGATG
Fasn sense	GGAGGTGGTGATAGCCGGTAT

Fasn anti-sense	TGGGTAATCCATAGAGCCCAG
Apoc1 sense	TCCTGTCCTGATTGTGGTCGT
Apoc1 anti-sense	CCAAAGTGTTCCCAAACTCCTT
Apoc2 sense	ATGGGGTCTCGGTTCTTCCT
Apoc2 anti-sense	GTCTTCTGGTACAGGTCTTTGG
Sh3bp5 sense	GCCTGGAGAGGATCTCAGATG
Sh3bp5 anti-sense	TCAGACGGACTCGTCGGTC
Lum sense	CTCTTGCCTTGGCATTAGTCG
Lum anti-sense	GGGGGCAGTTACATTCTGGTG
Fap sense	GTCACCTGATCGGCAATTTGT
Fap anti-sense	CCCCATTCTGAAGGTCGTAGAT
Timp1 sense	GCAACTCGGACCTGGTCATAA
Timp1 anti-sense	CGGCCCGTGATGAGAAACT
Twist1 sense	GGACAAGCTGAGCAAGATTCA
Twist1 anti-sense	CGGAGAAGGCGTAGCTGAG
Mmp1 sense	CCAGGGTGTTGGACTATGTTTG
Mmp1 anti-sense	CCCCGAGGAAGGTTCATCTTTA
Mmp2 sense	CAAGTTCCCCGGCGATGTC
Mmp2 anti-sense	TTCTGGTCAAGGTCACCTGTC
H2-Aa sense	TCAGTCGCAGACGGTGTTTAT
H2-Aa anti-sense	GGGGGCTGGAATCTCAGGT
Cd74 sense	AGTGCGACGAGAACGGTAAC

Cd74 anti-sense	CGTTGGGGAACACACACCA
H2-Ab1 sense	AGCCCCATCACTGTGGAGT
H2-Ab1 anti-sense	GATGCCGCTCAACATCTTGC

Supplementary Table S5. The list of reagents and antibodies used in this study.

**Antibodies**	**Identifier**	**Source**
APC anti-human CD36	Cat: 561822	BD Pharmingen
PE anti-human HLA-DRA	Cat: FAB4869P	R&D Systems
CCL5	Cat# MAB678	R&D systems
IgG	Cat# 1-001-A	R&D systems
IKZF1	Cat #14859	Cell Signaling
CEBPA	Cat # sc-365318	Santa cruz
CEBPD	Cat # sc-365546	Santa Cruz
β-tubulin	Cat# 2128	Cell Signaling
CCL5	Cat# 2988S	Cell Signaling
CD36	Cat# ab252922	Abcam
MIF	Cat# ab187064	Abcam
p65	Cat# ab32536	Abcam
SOX2	Cat# ab92494	Abcam
OCT4	Cat# ab181557	Abcam
STAT3	Cat# ab68153	Abcam
HLA-DRA	Cat# ab92511	Abcam

ACTA2	Cat #ab7817	Abcam
CD33	Cat # ab269456	Abcam
CD11b	Cat # ab133357	Abcam
FOXP3	Cat #ab20034	Abcam
CD4	Cat #ab183685	Abcam
MYH11	Cat # ab224804	Abcam
LUM	Cat # ab168348	Abcam
CXCL13	Cat #ab199043	Abcam
CD8	Cat # ab217344	Abcam
CXCR5	Cat #ab254415	Abcam
CD20	Cat #ab78237	Abcam
BUV395 anti-mouse CD8a	Cat# 563786; RRID: AB_2732919	BD Pharmingen
PE/Cy7 anti-mouse IFN-g	Cat# 505826; RRID: AB_2295770	BioLegend
FITZ anti-mouse GZMB	Cat# 515403; RRID: AB_2114575	BioLegend
BUV737 anti-mouse CD4	Cat# 612761; RRID: AB_2870092	BD Pharmingen
FITC anti-human CD33	Cat# 366619	BioLegend
APC anti-mouse/human CD11b	Cat# 101211	BioLegend
PE anti-human HLA-DR	Cat# 327007	BioLegend
Alexa Fluor® 647 anti-mouse/rat/human FOXP3	Cat# 320013	BioLegend
Alexa Fluor® 647 anti-mouse Ly-6G/Ly-6C (Gr-1)	Cat# 108418	BioLegend

APC-eFluor 780 anti-Mouse CD3	Cat# 47-0032-82; RRID: AB_1272181	Invitrogen
FITC anti-mouse CD25	Cat# 102005	BioLegend
Brilliant Violet 711 anti-mouse CD45	Cat# 103147; RRID: AB_2564383	BioLegend
Phospho-p38 MAPK (Thr180/Tyr182) (D3F9) XP Rabbit mAb	Cat# 4511; RRID: AB_2139682	Cell Signaling
**Buffers, chemicals, peptides, and recombinant proteins**		
BODIPY FL C16	Cat# D3821	ThermoFisher
BODIPY500/510 C1, C12	Cat# 3823	ThermoFisher
Oxidized LDL Uptake Assay Kit	Cat# 601180	Cayman Chemical
Human DiI High Oxidized Low Density Lipoprotein	Cat# 770262-9	Kalenbiomed
BODIPY FL LDL	Cat# L3483	ThermoFisher
DMEM	Cat# 11965118	GIBCO
RPMI 1640 Medium	Cat# 21875034	GIBCO
PBS	Cat# 20012-068	GIBCO
Recombinant human MIF	Cat# HY-P7387	MedChemExpress
OxLDL	Cat# 770252-7	Kalenbiomed
LDL	Cat# 770200-4	Kalenbiomed
a-Tocopherol	Cat# 30776	Cayman Chemical
SB203580	Cat# 13067	Cayman Chemical
ISO-1	Cat# HY-16692	MedChemExpress

Stattic	Cat# HY-13818	MedChemExpress
Sulfo-N-succinimidyl Oleate	Cat# SML2148	Sigma-Aldrich
Lipid Peroxidation Assay Kit	Cat# ab243377	Abcam
